# From Lab to Field: Role of Humic Substances Under Open-Field and Greenhouse Conditions as Biostimulant and Biocontrol Agent

**DOI:** 10.3389/fpls.2020.00426

**Published:** 2020-05-12

**Authors:** Keiji Jindo, Fábio Lopes Olivares, Deyse Jacqueline da Paixão Malcher, Miguel Angel Sánchez-Monedero, Corné Kempenaar, Luciano Pasqualoto Canellas

**Affiliations:** ^1^Agrosystems Research, Wageningen University and Research, Wageningen, Netherlands; ^2^Núcleo de Desenvolvimento de Insumos Biológicos para a Agricultura (NUDIBA), Universidade Estadual do Norte Fluminense Darcy Ribeiro, UENF, Rio de Janeiro, Brazil; ^3^Department of Soil and Water Conservation and Organic Waste Management, Centro de Edafolog a y Biología Aplicada del Segura (CEBAS)-Consejo Superior de Investigaciones Cient ficas (CSIC), Campus Universitario de Espinardo, Murcia, Spain

**Keywords:** humic acid, fulvic acid, foliar application, fertigation, circular economy, sustainable agriculture

## Abstract

The demand for biostimulants has been growing at an annual rate of 10 and 12.4% in Europe and Northern America, respectively. The beneficial effects of humic substances (HS) as biostimulants of plant growth have been well-known since the 1980s, and they can be supportive to a circular economy if they are extracted from different renewable resources of organic matter including harvest residues, wastewater, sewage sludge, and manure. This paper presents an overview of the scientific outputs on application methods of HS in different conditions. Firstly, the functionality of HS in the primary and secondary metabolism under stressed and non-stressed cropping conditions is discussed along with crop protection against pathogens. Secondly, the advantages and limitations of five different types of HS application under open-fields and greenhouse conditions are described. Key factors, such as the chemical structure of HS, application method, optimal rate, and field circumstances, play a crucial role in enhancing plant growth by HS treatment as a biostimulant. If we can get a better grip on these factors, HS has the potential to become a part of circular agriculture.

## Introduction

The function and application of biostimulants and biopesticides have garnered considerable interest due to their potential as environmentally sustainable resources for agricultural production. A number of national and international projects on biostimulant material have been launched in the framework of the circular economy by extracting the beneficial material from waste materials across different sectors of agriculture, livestock, water infrastructure, mining, and energy (Xu and Geelen, 2018). Notably, the projects BIO-FERTIL (Poland), BIOFECTOR (Germany), and HUMIC-XL (Netherlands) have highlighted the potential use of humic substances (HS) from waste material for plant growth, which can be a component of a local circular economy. To provide scientific evidences of the potential use of biostimulants, several reviews have been published recently (Calvo et al., 2014; du Jardin, 2015; Van Oosten et al., 2017; Abbott et al., 2018; Bulgari et al., 2019; Juárez-Maldonado et al., 2019; Pylak et al., 2019). In general, HS, seaweed extracts, beneficial microorganisms, and chitosan and protein hydrolases are listed in the mentioned review papers. While the chitosan and protein hydrolases are becoming popular as a biostimulant in the last decade (Drobek et al., 2019), utilization of HS, composed of humic (HA) and fulvic acid (FA), has been recognized as a long-run product since the 1980s (Calvo et al., 2014). The underlying function of HS as biological activation for plant growth has been strongly related to the chemical composition (e.g., functional groups), hydrophobicity, and flexible conformational structure of HS (Muscolo et al., 2013; Canellas and Olivares, 2017). Whereas a large number of scientific publications are related to the impact of HS in hydroponic assays and under growth chamber conditions (Nardi et al., 2000, 2018; Russell et al., 2006), reports on its potentiality in the field and under greenhouse conditions are less explored, mainly due to the variety of underlying factors in crop fields, including weather variability and climate fluctuations, soil type, and field management. For all these reasons, review reports on the practical application of HS in fields are scarce (Rose et al., 2014; Canellas et al., 2015b). The main focus of the present work is to (1) describe the mechanisms of the HS effect on plant growth, and (2) to illustrate the HS utilization under open-field and greenhouse conditions.

## Key Benefits of HS on Plant Growth

One of the major impacts of HS on plant growth is the reinforcement in nutrient uptake and the elongation of the lateral root growth, often recognized as “auxin-like effect,” which is a result of the induction of ATPase activity in the plasma membrane (Maggioni et al., 1987; Nardi et al., 1991; Pinton et al., 1992; Canellas et al., 2002; Quaggiotti et al., 2004; Zandonadi et al., 2007). The underlying mechanisms are generating a wider electrochemical gradient by ATPase induction and accelerating the nutrient uptake rate, which can also be confirmed by the overexpression of the transporter genes (Jindo et al., 2016; Zanin et al., 2018; Nunes et al., 2019). The availability of micronutrients such as iron can be improved with HSs, not only by chelation but also by promoting the root capability to uptake nutrients from the soil solution (Aguirre et al., 2009; Zanin et al., 2019).

Understanding the underlying mechanisms of plant response is a noteworthy keystone for the HS use in the field, and the first step would be a better understanding of the effect of HS on carbon and nitrogen cycles, which is related to primary metabolism (Canellas and Olivares, 2014; Olk et al., 2018; Canellas et al., 2019). HS also interferes with secondary metabolism by altering gene expression and changing the content of chemical compounds in plant cells, such as those related to the Krebs cycle, metabolism of nitrate and phosphorus, glycolysis, and photosynthesis (Roomi et al., 2018; Lotfi et al., 2018).

Historically, from the 1980s until the end of the 1990s, studies on the effect of HS on photosynthesis and ATP production were the major topics of research. A critical view of these works can be found in the previous papers of Nardi et al. (2002, 2009). Trevisan et al. (2011) found a high level of transcription of genes involved in primary metabolism in *Arabidopsis thaliana* and supported previous studies about the physiological effects of HS on plant metabolic pathways. Nardi et al. (2007) evaluated the impact of different HS on the enzymatic activities involved in glycolytic and respiratory processes of maize seedlings including glucokinase, phosphoglucose isomerase, PPi-dependent phosphofructokinase, and pyruvate kinase, as well as the activity of citrate synthase, malate dehydrogenase, and isocitrate NADP^+^ -isocitrate dehydrogenase. In the proteomic analysis conducted by Nunes et al. (2019), differences were detected in the maize seedling root proteins related to energy metabolism, cytoskeleton, cellular transport, conformation and degradation of proteins, and DNA replication. Thirty-four proteins were significantly more abundant in the seedlings treated with HA, whereas only nine proteins were abundant in the control. The main effect of HA was protective, mainly associated with increased expression of 2-cys peroxidase, putative VHS/GAT, and glutathione proteins (Nunes et al., 2019).

The transcriptome and proteome are more abundantly reported than metabolomics studies. The plant metabolome is the entirety of the small molecules present in the plant and can be regarded as the ultimate expression of its genotype in response to environmental changes (Fiehn, 2002). Aguiar et al. (2018) observed that the application of HA on sugarcane significantly decreased the concentration of 15 metabolites, which generally included amino acids. HA increased the levels of 40 compounds, which are associated with the stress response (shikimic, caffeic, hydroxycinnamic acids, putrescine, behenic acid, quinoline xylulose, galactose, lactose proline, oxyproline, and valeric acid), and this is aligned with up-regulation of the protein involved in redox homeostasis (Roomi et al., 2018).

Plant secondary metabolism produces a large number of specialized compounds that do not directly aid in the growth and development of plants but are required for the plant to survive in its environment and under biotic and abiotic stress. Salinity and drought are the most frequent stresses studied in fields and under greenhouse conditions (Ali et al., 2020). Several reports have been published on the impact of HS on the growth of pepper, common beans, rice, tomato, corn, sorghum, and cucumber under these stress conditions (Demir et al., 1999; García et al., 2012; Berbara and García, 2014; Rose et al., 2014; Prado et al., 2016; Van Oosten et al., 2017; Bulgari et al., 2019; Pinos et al., 2019; Ali et al., 2020). One of the underlying mechanisms of the impact of the HS is the interaction with auxin, jasmonic acid and abscisic acid by phytohormonal regulation in the root, which are well-known plant hormones for the stress of drought and salinity (De Hita et al., 2019; Ali et al., 2020). Another example is the synthesis of flavonoids, which are involved in the interception of ultraviolet (UV) as an adaptive mechanism preventing UV in plant physiology (Hollósy, 2002). HA could induce the activity of the first enzyme in the phenylpropanoid pathway at the level of gene expression, similarly to other studies in which phenylpropanoid synthesis has been enhanced by fungal elicitors and hormones (Schiavon et al., 2010; Lewis et al., 2011).

The increase in phenolic compounds is another typical plant response to HA treatment (Ertani et al., 2011). During the progress of the domestication of cultivated plants over 10,000 years, the bitter and astringent taste from phenolic compounds, which often produced in the phenylpropanoid pathway of the secondary metabolism, has been gradually eliminated, resulting in the reduction of the natural plant protection against stress (Wink, 1988). The foliar application of HA improves this ancient mechanism reducing plant infection (Olivares et al., 2015) as well as enhancing plant protection (Hernandez et al., 2014).

Finally, HS is involved in the enhancement of plant protection against infestation. Joshi et al. (2014) present the list of pathogens and pests controlled through vermicompost application, highlighting that the main chemical components of the vermicompost belong to HS. There are four approaches by which HS can contribute to the plant defense mechanisms under field and greenhouse conditions: (1) enhancing the soil microbial activities that play as biological control agents, such as *Trichoderma* (McLean et al., 2012; Motta and Santana, 2013; Mohamadi et al., 2017); (2) direct interaction with plant pathogen (e.g., Nematodes, Late blight) (Zaller, 2006; Seenivasan and Senthilnathan, 2018; D’Addabbo et al., 2019; Liu et al., 2019); (3) physical protection for beneficial microbes, such as UV protection (Bitton et al., 1972; Muela et al., 2008; Kaiser et al., 2019); (4) enhancing plant antioxidant defense system against pathogen by modulating chemical compounds (e.g., phenols) and enzymes (e.g., phenylalanine ammonia-lyase) (Kesba and El-Beltagi, 2012; Olivares et al., 2015).

## Mode of Application in Fields

The functions of HS for the enhancement of plant growth widely differ depending on the application mode, plant stage, and its rate, which will be discussed in the subsequent sections. Basically, there exist five application types of HS in the field (Erro et al., 2016; Ekin, 2019).

### Direct Application in Soil (Liquid Status)

Researches and farmers adopt the direct use of HSs as an aqueous suspension. The effect of the direct application of liquid status has been demonstrated on the growth of different crops such as Lettuce (*Lactuva sativa*) and Grape rootstocks (*Vitis vinifera* L.) (Supplementary Table S1). Comparative advantages of liquid formulation include the possibility to combine with other inputs such as chemical fertilizer or beneficial microorganisms and adaptability to agricultural machinery for the implementation. Application time, depending on the plant development stage, must be considered.

### Direct Application in Soil (Solid Status)

The solid-state application of HSs has been less explored for implementation in the field when compared with liquid formulations. The main agricultural applications of HSs in the form of powder or granules are soil amendments and organo-mineral fertilizers that require the highest dose per plot (Supplementary Table S1). The solid application brings a problem of uniform distribution of aqueous dispersion after dissolution on rhizosphere, gradient concentration, and re-sedimentation of HA on soil solution. Despite the difficulty of obtaining uniform HS aqueous suspension at the optimal doses, different rates of solid HS application had shown a direct positive effect on plant stimulation or soil physicochemical properties (Supplementary Table S1). Powder HA applied to soil at a rate of 75 g m^–2^ increased yield of thyme (*Thymus vulgaris* L.) and quality of essential oil (Noroozisharaf and Kaviani, 2018). In the same study, the highest dose of HA powder (100 g m^–2^) improved nutrient content in leaves via positive modulation of nutrient transport through the chelation and stimulation of microbial activity by HS interaction. Undoubtedly, solid forms as powder or granules will be suitable in the future since the transport operation can be economically prohibitive for liquid HSs. However, a high volume of HS products is required for large-scale farming. Future research on the technology of on-farm solubilization of solid forms as stable final products will be demanded.

### Foliar Application

Since the 1940s and 1950s, scientific research on the beneficial impact of foliar application has been explored (Tanou et al., 2017). There exist two theories to explain how exogenous inputs via foliar application are delivered to plant cell tissue, once they reached leaf surface: (1) transfer into leaf tissues via transcuticular penetration (Smilkova et al., 2019); or (2) penetration through leaf stomata (Tejada et al., 2016). Many authors report that micronutrient contents are increased by HS rather than macronutrient in field level (Fernández-Escobar et al., 1996; Çelik et al., 2011; Fatma et al., 2015; Balmori et al., 2019). After foliar treatment, nutritional parameters of polyphenol content and antioxidant activity to determine the quality of fruit are improved (Tarantino et al., 2018). In practice, liquid compost extracts, fully enriched with HS, represent a cost-effective tool to conduct foliar application (Zandonadi et al., 2013; Berbara and García, 2014). A wide range of plants have been tested with HS application under open-field conditions, such as garlic (Balmori et al., 2019), common beans (Kaya et al., 2005; Souri and Aslani, 2018), wheat (Zhang et al., 2016; Ahmad et al., 2018; Bezuglova et al., 2019), fenugreek (Ibrahim, 2019), tomato (Olivares et al., 2017), asparagus (Tejada and Gonzalez, 2003), maize (Canellas et al., 2015a) and citrus tree (Hameed et al., 2018). Foliar application is frequently reported in calcareous soil conditions where nutrient uptake, especially iron, is limited due to precipitation (Çelik et al., 2011; Souri and Aslani, 2018; Bezuglova et al., 2019). Foliar spray application is limited to suitable climate conditions, since high temperature and windy and rainy days are not recommended. High application rates provoke leaf burning as water evaporates and salts remain on the leaves, especially at high temperature (Fageria et al., 2009). The developing stage has to be considered since foliar application cannot be conducted after flowering in rice production, which could cause spikelet discoloration. Crop responses to foliar application are unlikely positive when there is nutrient deficiency in the soil (Fageria et al., 2009). Taking all together, the impact of foliar-applied HS is less consistent than those observed when applied on the root, where HS is exposed to a more stable condition (De Hita et al., 2019).

### Fertigation

Fertigation is extensively expanding over the world, especially in semi-arid and arid regions where water scarcity is an issue (Fallahi et al., 2017). García-Gaytán et al. (2018) widely describe the potential of different biostimulant materials used in fertigation. After the concentration of HS in rhizosphere increases by the irrigation, two contributions of HS to plant growth are presumably proposed: (1) straightening out soil fertility, which makes nutrient more available; (2) directly reaching out plant cell walls on the root surface so that plant can take up nutrients (Olaetxea et al., 2018). Regarding agronomic outcome in practice, Suman et al. (2016, 2017) showed the impact of the combined application of chemical fertilizer and HA in fertigation on productivity on capsicum and tomato under open-field conditions, concluding that HA could replace up to 20% of fertilizer. Selladurai and Purakayastha (2016) used a similar combination of liquid fertilizer by using the pedal-operated sprayer in soil in the open field, and they improved N, P, and K use efficiencies by 16.4, 9.3, and 18.3%, respectively. Water use also can be saved by the humic application (Selim and Mosa, 2012; Alenazi et al., 2016). The mode of fertigation has to be adjusted based on the type of crop. Selim et al. (2009) highlighted that subsurface drip irrigation method has a highly significant effect on potato tuber yield rather than surface drip irrigation, due to maintenance of optimum soil moisture content in the root zone in an Egyptian sandy soil. However, no effect was found in banana seedling with the drip irrigation with HS in tropical soil (de Melo et al., 2016), implying that crop and soil type have to be taken into account. A multiple-option of HS application, combining the use of solid HS at pre-sowing moment prior to fertigation with HS, can be useful to mitigate adverse environmental conditions (Smoleñ et al., 2017), or the use of wastewater for fertigation with HS incorporated into soil for saving water resources (Masciandaro et al., 2014).

### Immersion

A limited number of works are reported on the seedling with the immersion method under field and greenhouse condition (Bettoni et al., 2016a, b; Gemin et al., 2019). This method is commonly used in hydroponic and growth chamber conditions (Supplementary Table S1).

## Benefits and Limitations of Hs Application in the Field

Proper implementation of HS in field conditions is an essential point for experiment design. Several works report a comparative study of different applications (Supplementary Table S1). Waqas et al. (2014) compared three application modes (foliar spray, soil application, and immersion) for mung bean. They concluded that no significant differences were observed across different applications. A similar result was reported by Karakurt et al. (2009) on pepper comparing between foliar spray and soil application. In contrast, other reports showed that foliar spray performed higher yield than soil application in tomato (Yildirim, 2007), maize (Tejada et al., 2016), almond (Saa et al., 2015), and sugarcane (Da Silva et al., 2017). An ideal implementation would be combined applications rather than a single application method, which was demonstrated in Bettoni et al. (2016b) with higher nutritional quality and yield of onion.

**FIGURE 1:**
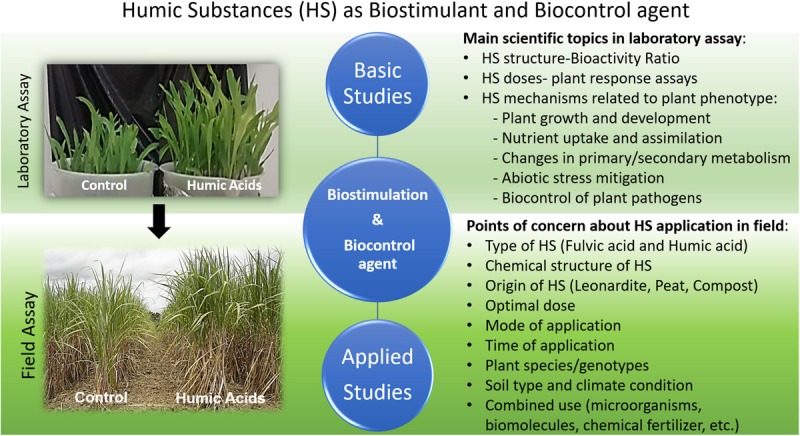
Advantages and limitations of humic substance application under open-fields and greenhouse conditions.

It is noteworthy that the positive effect of HS application on plant growth is not always guaranteed. The points of concern about the HS application are listed in Figure 1. In particular, the chemical structure of HS, optimum application rate, and the mode of use play a crucial role in performing a visible outcome on the ground. At first, finding out an optimal dosage is an essential process, and this is changeable with application mode and plant type. Some specific plants such as lettuce (*L. sativa*) from *Asteraceae* family and *Arabdopsis thaliana* from *Brassicacea* family are more sensitive to the change in the concentration of HS and application mode (Rodda et al., 2006; Dobbss et al., 2007; Hernandez et al., 2013). Secondary, the type of HS is a vital point, which is related to the chemical structure and molecular size of HS. The interaction between the chemical composition of HSs and bioactivity was studied (Canellas et al., 2009; Aguiar et al., 2013; Martinez-Balmori et al., 2014) and the importance of hydrophobic/hydrophilic ratio is a key factor as a suitable indicator to predict bioactivity based on their chemical properties. This ratio is prominently high in HA rather than FA due to the enrichment of the aromatic carbon group. Also, similar or even better crop responses have been achieved by HSs derived from compost rather than from leonardite, peat, or other pedogenic stable organic matter reservoirs (Ayuso et al., 1996). Another factor is the chemical variation due to different extraction techniques and nutrient enrichment processes (Hartz and Bottoms, 2010). In line with this study, Chen et al. (2004) concluded that soil application of commercial humic products at typical rates (2 to 3 kg ha^–1^) is ineffective in promoting significant agronomical response to different crops under an open-field condition.

Furthermore, Chen et al. (2004) highlighted that the recommended dose for commercial HS product is at least 10 times smaller than required to stimulate plant growth under laboratory and greenhouse assays (75 mg L^–1^, equivalent to 50 kg ha^–1^). Regarding soil types, Pylak et al. (2019) report that HSs are not particularly effective in reducing the solubility of heavy metals in acidic soils. Using commercial HA products in combination with liquid fertilizers, Hartz and Bottoms (2010) mentioned that a positive crop response was found only in soil with low organic matter content. Also, suitable application time is a concerned issue. While the use of HS at the early developing stage usually enhances the root elongation, sugar content, grain weight, and fruit size increase at a late vegetative stage (Canellas et al., 2015b).

## Conclusion

HS application originally from wastes as a biostimulant for plant growth is a beneficial and eco-friendly approach, and it fits into the concept of circular economy focusing on the conversion to a new resource. Plant anatomical and biochemical changes in the root system by HS are the main factors responsible for increased nutrient uptake, although the increase in the nutrient availability through chelation is another HS contribution to plant growth. The hydrophobicity/hydrophilic ratio is a useful indicator to understand the chemical structure of HS and to estimate the effect on plant growth. Although different dose ranges of HS application in field and laboratory condition are suitable, it is recommendable to conduct a preliminary test to find an optimum rate considering crop type, soil properties, and application mode.

## Author Contributions

All authors contributed to the study conception, design, data collection, analyses, and manuscript preparation. KJ, FO, DM, LC, wrote the article. KJ, FO, MS-M, CK, and LC supervised and completed the writing. All authors read and approved the final manuscript.

## Conflict of Interest

The authors declare that the research was conducted in the absence of any commercial or financial relationships that could be construed as a potential conflict of interest.

## Supplementary Material

TABLE S1 Effect of humic substance with different application modes on plant growth under different experimental conditions: Field trial (FD); Growth chamber (GC); Greenhouse (GH) Hydroponic (HP).
