# Supplementary material for: From Lab to Field: Role of Humic Substances Under Open-Field and Greenhouse Conditions as Biostimulant and Biocontrol Agent
Source: Front Plant Sci. 2020 May 12;11:426. doi: 10.3389/fpls.2020.00426 (PMC7247854; doi:10.3389/fpls.2020.00426)
Supplement: TABLE S1 — Effect of humic substance with different application modes on plant growth under different experimental conditions: Field trial (FD); Growth chamber (GC); Greenhouse (GH) Hydroponic (HP). [file Table_1.pdf]

Table Caption:

Table S1. Effect of humic substance with different application modes on plant growth under different experimental conditions: Field trial (FD); Growth chamber (GC); Greenhouse (GH) Hydroponic (HP).

Table 1. Effect of humic substance with different application modes on plant growth under different experimental conditions: Field trial (FD); Growth chamber (GC); Greenhouse (GH) Hydroponic (HP).

**Application Mode: Direct application in Soil (liquid status)**							
**Crop**	**Type of humic substances**	**Specification**	**Dosage**	**Condition**	**Effects**	**Observation**	**Reference**
Soybean (*Glycine max*)	Leonardite potassium humate. (Powhumus, Humintech Ltd., Germany)	C 34.9%, H 3.89%, N 0.72%, S 0.06%, Fe 0.45%	35, 75, and 150 μmol pot^{-1} iron-humic material. (600 g pots) or ~ 58.33, 125 and 250 μmol L^{-1} ^{57}FeEDDHA (50 μmol pot^{-1}), as a positive control, and L (providing 8.9 μmol Fe pot^{-1})	GC	The ^{57}Fe-NFs supply Fe to the plants (root to shoot transport). Slow and continuous iron release of these ^{57}Fe-NFs confirms their long-term effect in providing iron in calcareous conditions. Remain available to the plant requirements	Eco-friendly iron-humic nanofertilizer synthesis for the prevention of iron chlorosis	Cieschi et al., 2019
Soybean (*Glycine max*)	The humic acid (HA) commercial product of Sigma-Aldrich®	pH in water of 9.8; C 376 g kg^{-1}, N 7.6 g kg^{-1}, P 0.3 g kg^{-1}, K 6.3 g kg^{-1}, Ca 5.9 g kg^{-1}, Mg 0.3 g kg^{-1}, S 4.4 g kg^{-1}, Fe 1.1 g kg^{-1}, Cu 40 mg kg^{-1}, Mn 15 mg kg^{-1} and Zn 22 mg kg^{-1}	5, 10, 50 and 100 mg L^{-1} C_{HA}	GH	Effects of HA on soil attributes, soybean growth and nutritional status rely on HA concentration, soil texture and P source used	---	Rosa et al., 2019
Blueberry (*Vaccinium corymbosum* L.)	Biosolve (Oikos Chile Ltda.), commercial product containing humic acid (derived from leonardite shale)	humic acid 70%, fulvic acid 15%, and K_2O 10% w/w	15000 mg L^{-1} C_{HA}	FD	Total polyphenols and ferric reducing antioxidant power (FRAP) in fruit increase	Combined use with microbial inoculant	Schoebitz et al., 2019
Maize (*Zea mays* L.)	Humic acids (HA) extracted of vermicompost	C 47%, H 5.1%, N 3.1%, O 43%.	50 mg L^{-1} C_{HA} (3,5 mM C)	HP in GC	Plant growth promotion and increased expression of the 2-cys peroxidase, putative VHS/GAT, and glutathione proteins	---	Nunes et al., 2019
Maize (*Zea mays* L., var UENF 506)	Humic acids isolated from vermicompost of cattle manure.	HA: 48%, 3.8%, 5.0% and C, N, H, and O 43.2%. Ash content was low (< 0.5%)	20 mg L^{-1} C_{HA} extracted from earthworm compost or 10^{-4}, 10^{-6} or 10^{-8} M *N*-isopropyl decanamide	HP in GC	Both N-isopropyl decanamide and humic acids enhanced nitric oxide accumulation during lateral root emergence	---	Zandonadi et al., 2019

Maize (*Zea mays* L. Dekalb 7815)	Humic acids isolated from Earthworm compost (10:1, v/v)	HA solution was prepared by solubilizing HA powder in 1 mL of 0.01 M NaOH, followed by pH adjustment to 6.5 with 0.1 M HCl	3.5 mM C L^{-1} (n = 4 pots per treatment with 18 seedlings per pot)	HP in GC	The increase on exudation yield induced by humic acids enhanced the release of a plethora of chemical compounds to root interface. The humic substances influence population size and microbial community structure	---	Canellas et al., 2019a
Maize *(Zea mays)* and Sugarcane (*Saccharum* spp.)	Earthworm compost (10:1, v/v)	The HA solution was prepared by solubilizing HA powder in 1 mL of 0.01 M NaOH, followed by pH adjustment to 6.5 with 0.1 M HCl	48 mg L^{-1} C_{HA}	HP in GC	The biostimulant induced changes in the metabolite fingerprints of maize and sugarcane seedlings. 22 putative biomarkers associated with the biostimulant-treated plant-phenotype were identified	Combined use with endophytic diazotrophic bacteria (*H. seropedicae* and *G. diazotrophicus)*	Canellas et al., 2019b
Maize (*Zea mays* L. var. UENF 506-11)	Humic acids isolated from vermicompost of filter cake, which is a typical product of the sugar industry in the north of Rio de Janeiro State, Brazil.	Exhibited low carbon 46%, high nitrogen 5.7% and high oxygen content 45% compared with HA from pedogenic sources	2 mM L^{-1} C_{HA}	HP in GC	Inoculation activated the extracellular H^+ flux membrane potential of and aquaporin overexpression. Nitrate transporters were repressed by the inoculants	Combined use with *H. seropedicae*	De Azevedo et al., 2019
Maize (*Zea mays* L.)	Vermicomposting composed by cow manure and lignocellulose residues from flock cotton production provided by textile company (ARS S/ A RJ Brazil)	Relative yield (%) and dimensionless index released by humic acids (0-100). Carbohydrate: 2.3 - 10.2 N: 2.3 – 7.2	3.5 mM L^{-1} C_{HA}	HP in GC	Higher bioactivity found in humic components isolated from vermicompost with no or low addition of cotton residues	---	De Aquino et al., 2019
Bean (*Phaseolus vulgaris* L.)	Fulvic acid (LX7®, MTS Environmental Inc. Centralia, ON) or humic acid (Plant XL®, Alpha-Agri, Bluevale, ON)	Not specified	Fulvic fertilizer at 5, 10 (recommended rate), 20, 40, or 80 L ha^{-1} (i.e., 1/2×,1×,2×,4×,and 8× the recommended rate). Humic fertilizer at 2.5 L ha^{-1} (the recommended rate)	FD	Twenty fulvic acid field trials and 15 humic acid field trials indicate that these fertilizers were ineffective, as plant vigor, height, 100-seed weight, and yield were similar to a control treatment		Mahoney et al., 2017

Maize (*Zea mays* L.)	HA isolated from the following green composts: 1. Artichoke (HA-CYN); 2. artichoke/fennel (HA-CYNF); 3. Tomato (HA-TOM), 4. Cauliflower (HA-CAV).	HA-CYN (%):C 29.10, N 2.52, H 3.79, C/N 13.44, H/C 1.57. HA-CYNF (%): C 35.62, N 3.00, H 5.29, C/N 14.14, H/C 1.78. HA-TOM (%): C 38.36, N 2.91, H 4.9, C/N 15.24, H/C 1.53. HA-CAV (%): C 32.50, N 2.62; H 4.26, C/N 14.26, H/C 1.57.	25, 50 and 100 mg L^{-1} C_{HA}	HP in GC	Bioactivity from green composts related to their composition/conformational structure in solution	---	Monda et al., 2018
Wheat (*Triticum aestivum* L.)	Not specified	Not specified	750 or 1500 mg L^{-1} C_{HA}	HP	HA application mitigate negative effects of Cd stress induced, on parameters water content, osmotic potential, disrupted chlorophyll fluorescence trait sand higher proline content.	with/without HA treatment under Cd toxicity	Ozfidan-Konakci et al., 2018
Sugarcane (*Saccharum* spp.)	Earthworm compost	100 mL of each bacterial suspension in 800 mL of HA solution at pH 6.5 (bacteria + humic acid).	50 mg L^{-1} C_{HA}	GH	Antioxidant/osmoprotective agents produced in inoculated plants. HA increased metabolic response of bacteria inoculation.	Combined use with *H. seropedicae* and *G. diazotrophicus* bacteria endophytes	Aguiar et al., 2018
Banana (*Musa* spp.)	Commercial product containing HA (potassium humate) from M/s. Neyveli Lignite Corporation	The humic acid content was 4% and the pH 8.5. Natural by-product derived from lignite.	Concentrations (0.04%, 0.08%, 0.2% and 0.4%)	GC and GH	Control root-knot nematode in vitro and growth of banana plantlets in terms of number of leaves, pseudo-stem height, girth, weight, root length and weight were noticed in humic acid treated pots.	---	Seenivasa and; Senthilnathan, 2018
Wheat (*Triticum aestivum* L.)	Commercial product of Sigma-Aldrich®	pH in water of 9.8; C 376 g kg^{-1}, N 7.7 g kg^{-1}, P 0.3 g kg^{-1}, K 6.3 g kg^{-1}, Ca 5.9 g kg^{-1}, Mg 0.3 g kg^{-1}, S 4.4 g kg^{-1}, Fe 1.1 g kg^{-1}, Cu 39.9 mg kg^{-1}, Mn 14.8 mg kg^{-1}, and Zn 21.8 mg kg^{-1}	5, 10, 50, 100 mg L^{-1} C_{HA}	GH	The right combination of HA and Araxá phosphate rock (APR) leads to wheat growth and nutrient uptake equivalent to those found for single superphosphate (SS).	---	Rosa et al., 2018
Maize (*Zea mays* L., hybrid P6875 from DEKALB)	Vermicompost was prepared using sugarcane filter cake and ground sugarcane from a	C 48%, N 3.4%, H 2.8%, O 46%	3.5 mM of carbon of the HA.	HP in GC	HAs interfere with nutrient sensing because target of rapamycin (TOR) expression was induced independently of amino acid, sugar, or organic acid concentrations found in leaf	---	Canellas et al., 2018

	commercial factory.				and root tissues.		
Sugarcane (*Saccharum* spp.)	Humic acids were extracted of vermicompost	Humic acids were extracted from 10 g of vermicompost with 100mL of 0.1 mol L^{-1} NaOH solution under N_2 atmosphere	20 mg $L^{-1}C_{HA}$	GH	The antioxidant enzymes remained higher after rehydration only in plants treated with HA. Plants treated with HA and PGPB+HA exhibited increased net photosynthesis than plants treated with PGPB.	Combined use with plant growth promoting bacteria and humic substances under water stress.	Aguiar et al., 2016
Soybean (*Glycine max*)	Organomineral fertilizer, enriched with HSs (humic extract from peat).	Density 1.1 g mL^{-1}; pH in $CaCl_2$ of 9.0, N 1.1 g L^{-1}, K 17.0 g L^{-1}, B 14.8 g L^{-1}, Zn 35.0 g L^{-1}, cation exchange capacity of 850 cmolc dm^{-3}, organic carbon 35.0 gL^{-1}, total humic extract 108.0 g L^{-1}, humic acid 58 g L^{-1}, and fulvic acid 50.0 g L^{-1}	1000, 2000, 4000 and 8000 mg L^{-1}	GH	Positive response to the doses, with the most efficient one under water stress being 6.5 mL dm^{-3} and HSs favored the uptake of micronutrients	Soybeans Grown Under Water Stress	Prado et al., 2016
Tomato (*Solanum lycopersicum* L. cv. Micro-Tom)	Isolated from vermicompost	HA (O% = 100 – C% – H% – N%) on an ash-free basis, and the elemental composition was: C 46%, N 5.7%, H 3.0%, O 45%	48 mg L^{-1} C_{HA}	GC	High transcript accumulation of *LePT2* was observed in roots treated with humic acids at both P concentrations	Combined use with low or high P concentrations (10 or 100 mg kg^{-1} KH_2PO_4)	Jindo et al., 2016
Maize (*Zea mays* L., 'DKB 789')	The HA was obtained from filter cake sugarcane vermicompost	Not specified	20 mg L^{-1} C_{HA}	GH	Microorganisms plus humic acid positively stimulated root and shoot weight by 17 and 22%, respectively	Combined use with P-solubilizing microorganisms (PSMs)	Giro et al., 2016
Rice (*Oryza sativa* L. cv. Nipponbare)	Vermicompost humic acids	C 52.22%, H 7.58%, O 36.94%, N 1.85%, S 1.41%.	5, 10, 15, 20, 40, 80 and 100 mg L^{-1} of organic carbon (pH 5.8)	HP in GC	Action of VCHA on the root development of non-stressed or PEG-stressed rice plants involves modulation of reactive oxygen species (ROS) accumulation in roots	Unstressed and PEG-stressed rice plants associated with humic acid application	García et al., 2016a
Tomato (*Solanum lycopersicum* L.)	Extracted from vermicomposted cattle manure	Organic matter (dry weight basis) 36.2 g kg^{-1}, total humic substances 25.8 g kg^{-1}, humic acids 12.8 g kg^{-1}, fulvic acids13.0 g kg^{-1}, pH 8.67, electrical	20 mg L^{-1} C_{HA} (pH 7.0). Humates were diluted 20-fold in distilled water before application.	GH and FD	Plant substrate application at nursering condition increased growth rates of tomato plants	Combined use with *Herbaspirillum seropedicae*	Olivares et al., 2015

		conductivity 11.7 mS cm^{-1}, total N 1.4 g kg^{-1}, total P (as P_2O_5) 12.6 g kg^{-1} and 3% ash					
Rice (*Oryza sativa* L.)	HA was isolated and purified from three-month-old cattle manure	C 52.22%, H 7.58%, O 36.94%, N 1.85%, and S 1.41%.	20, 40 and 80 mg L^{-1} C_{HA})	HP in GC	Protective effect through signaling mechanism independent of ABA, and regulation of OsTIPs genes on plant root system	---	García et al., 2014
Spring onion seed cv. 'Paragon' and Tenet ®	Humic acid pellets Agrolig™ and Humic acid liquid Supa Humus™	Pellets (86% organic matter; 75% humic acid, Agrichem, Australia, 2011) (12% liquid humic acid, Agrichem, Australia, 2011)	450 mg L^{-1} (10 ml/10 cm furrow) using a solution of 3L product/1000 L water	GH	Application strategy for the use of a commercial formulation of *T.atroviride* C52 in an integrated white rot management program for onions is proposed	Combined use with *Trichoderma atroviride* Karsten strain C52	Mclean et al., 2012
Grape rootstocks *(Vitis vinifera* L.)	Commercial products of humic acid (liquid and granules)	Not specified	2000 and 4000 mg plant	GH	The improvement grape yield by increasing the content of antioxidant compounds and the specific activities of antioxidant enzymes	---	Kesba and El-Beltagi, 2012
Gerbera (*Gerbera jamesonii* L.) cv. 'Malibu'	Humic acid prepared from leonardite was purchased from a Chinese company.	Containing C 61.2%, N 3.13 g and P 2.89 g per kg dry matter	100, 500, and 1000 mg L^{-1} C_{HA}	GH	Humic acid as nutrient uptake enhancer instead of using high nutrients concentration. Promising in hydroponic production of cut flowers specially gerbera to benefit from improving nutrients uptake	---	Nikbakht et al., 2008
Crucifer (*Arabidopsis thaliana* L.)	HA, FA and HM extract isolated from a representative Oxisol (Sombrihumox)	Average of seven Oxisol. Humic acids: C 507 g kg^{-1}, H 60 g kg^{-1}, N 55 g kg^{-1}, O 571 g kg^{-1}, Ash 80 g kg^{-1}. Fulvic acids: C 376 g kg^{-1}, H 55.7 g kg^{-1}, N 72 g kg^{-1}, O 693 g kg^{-1}, Ash 164 g kg^{-1}. hexanic–methanolic: C 135 g kg^{-1}, H 24 g kg^{-1}, N 25 g kg^{-1}, Ash 22 g kg^{-1}. (pH 5.8).	Doses of 0, 3, 30 and 300 mg L^{-1}. Organic matter fractions of the seven Oxisol at 40, 100 and 100 mg L^{-1} for HA, FA and HM, respectively	HP	Humic fractions were able to change the root development, improving the number of lateral roots and their development	---	Dobbss et al., 2007

Maize (*Zea mays* L.)	HA isolated from a volcanic soil	C 39.48, N 2.26, O 3.13, pH 7.2	0, 0.5, 1, and 5 mg L^{-1} C_{HA}	HP in GC	A better knowledge of the relationship between molecular structure of soil humic matter and plant activity may be of practical interest in increasing carbon fixation in plants	---	Nardi et al., 2007
Lettuce (*Lactuca sativa* var - Regina)	Humates isolated from cattle manure vermicompost	Not specified	0 - 100 mg L^{-1} C	GH	25 mg L^{-1} of C dose increased root mass as well as root area and total length. Also, increase of lateral roots and ATP hydrolysis microsomal root fraction	---	Rodda et al., 2006
Maize (*Zea mays*)	Humic acids (HAs) isolated from cattle manure earthworm compost	Elemental composition analysis of HA, the values for total carbon, oxygen, nitrogen, hydrogen, and ash were 48.5%, 42.2%, 3.2%, 5.6%, and 0.51% (on a dry weight basis), respectively	20, 40, or 80 mg dry weight L^{-1} HA extracted from earthworm compost	HP in GC	HA enhance root growth, marked proliferation of sites of lateral root emergence. They also stimulate the plasma membrane H^+-ATPase activity, apparently associated with an ability to promote expression of this enzyme	---	Canellas et al., 2002
Maize *(Zea mays)* and Coffee *(Coffea arabica)*	Humic acids extracted from sewage treatment plant sludge (AHL) and humic acids extracted from vermicompost (AHV).	AHL: C 54 %, H 7 %, N 4.9 %, ash 0.74 %.<br><br>AHV: C 48.5 %, H 5.57 %, N 3.2%, ash 0.51%.	20, 40 and 80 mg L^{-1} (pH 5.5)	HP in GC	Root surface area stimulation was observed in both species cultivated with both HA, showing an optimal concentration around 40 mg L^{-1}	---	Façanha et al., 2002
**Application Mode: Direct application in soil (solid status – granule or powder)**							
**Crop**	**Type of humic substances**	**Specification**	**Dosage**	**Condition**	**Effects**	**Observation**	**Reference**
Forage Sorghum (*Sorghum bicolor* L.)	Jasmonic acid (JA) provided by Shanghai Yuanye Biotech. Co., Ltd., Shanghai, China; Humic acid (HA) Dalian Vic Co., Ltd., Dalian, China	C 55%, N 4.87%, P 0.01%, K 11.21%, Ca 0.50%, and Mg 0.22% (pH 7.08)	JA (0, 5, and 10 mM) and HA (0, 3, and 6 g HA kg^{-1} soil)	GH	The combined application of JA and HA application management can be used in salt-affected soil to sustain forage growth and to increase yield and productivity of crops under grown in saline soil	---	Ali et al., 2019

Watermelon (*Citrullus lanatus*)	The solid humic substances, herein HS (Novihum Co., Dresden, Germany)	Humic acid 56.7%, humin 24.1% and fulvic acid 0.7%	2500 mg L^{-1} (5 t ha^{-1})	FD	HS increased triploid watermelon yield by 73%. Under deficit irrigation (115%) and increased total marketable yield (20%). Also increased SOC (24%)	Plants under water stress conditions, with or without humic substances	Qin and Leskovar, 2019
Potato (*Solanum tuberosum* L.)	Agro-Lig (Altintar Chemicals Company, Turkey) crude humic acids (derived from leonardite)	Total humic acid 85%, OM 75%, pH 3.5–5.5, max moisture 22%, 0.5%, Fe 0.5%, Mg 0.5%, Ca 3.0%, Na 0.3%, Mg0.02%, Fe 0.0003%, K 0.07%, Ti 0.02%, Ba 0.03%, and B 0.01%, Co 0.0002% dry matter basis.	In granule form at 100, 200 and 300 mg L^{-1} (200, 400, and 600 kg ha^{-1})	FD	Plant growth, tuber size and weight nutritive value and quality	Combined use with *Bacillus megaterium* and *Bacillus subtilis*	Ekin, 2019
Rice (*Oryza sativa*)	HA extracted from lignite (purchased from MACKLIN Ltd.).	The carbon, hydrogen, nitrogen, oxygen, total acidity, carboxyl COOH, and phenolic OH were 57.19% (w/w), 3.87% (w/w), 1.11%(w/w), 37.83%(w/w), 5.47mmol g^{-1}, 1.67 mmol g^{-1}, and 3.8 mmol g^{-1}, respectively.	4000 and 8000 mg L^{-1} (4 and 8 g kg^{-1} soil)	GH	HA inhibited the accumulation of Se and Cd in rice seedlings. Compared with adding Se (or HA) alone, application of Se mixed with HA might be a more effective way to produce Se-enriched and Cd- efficient crop in Cd-contaminated soil	Agricultural soils were treated with different concentrations of HA and selenium (Se) and cadmium (Cd)	Zhang et al., 2019
Sea Orache (*Atriplex halimus* L.)	Commercial humic substances extracted from leonardite (highly oxidized lignite). Humintech GmbH (Germany) PerlHumus® granules	Composed of humic acids (60%), pH 5.0–6.0	Dose of 120 mg L^{-1}, equal to 360 kg ha^{-1}, and doubled to 240 mg L^{-1}, equal to 720 kg ha^{-1}	GH	Lixiviation of Cu from mine tailings was lower when humic substances were added, with an average total load of 0.157–0.166 mg kg^{-1} compared to 0.251 mg kg^{-1} without added humic substances	Prevention from leaching of metals, seeds germinated directly in mine tailings (MT)	Tapia et al., 2019
Bell pepper (*Capsicum annuum* cv. Revolution)	Lignite-derived solid humic substances (Novihum Co., Dresden, Germany)	Composition of fulvic acid 0.7%, humic acid 56.7% and humin 24.1%, with total C 65.8% and total N 3.5%	Rate of 5 t/ha (2500 mg L^{-1}), incorporating with surface field soil (0–20 cm)	FD	HS had long-term positive influences on soil microbial activity through improvements in soil organic carbon content, which was also positively correlated with bacterial population	---	Qin et al., 2019

Garden thyme (*Thymus vulgaris* L. German cultivar 'Deutscher Winter')	Humic acid was purchased from the Kimia Pars Shayankar Company, Tehran, Iran. (In powder).	Humic acid containing N 1.3%, C 62.2%, O 36.8%, and H 2.4%	250, 375, 500 mg L^{-1} (50, 75, 100 g m^{-2}. Soil mixture depth was 30 cm)	GH	Humic acid could positively change nutrients uptake, essential oil content and its major constituents in *T. vulgaris*	---	Noroozisharaand Kaviani, 2018
Wheat (*Triticum aestivum* L.)	Plant-derived humic acid (PDHA) and coal-derived humic acid (CDHA)	PDHA: N 1.25%, C 53.48%, H 3.22%. CDHA: N 1.42 %, C 52.31%, H 3.15%.	25 and 50 mg L^{-1} (50 and 100 kg ha^{-1})	FD	Spike weight increased by 19% and 15% (clayey loam soil); 26% and 11% (sandy loam soil) with application of PDHA (50 and 100 mg/kg), respectively. Grain yield an increase of 21% (PDHA) and 11% (CDHA)	---	Khan et al., 2018
Pepper (*Capsicum annuum* L. cv. Revolution)	Lignite-derived HS	C 65.8 %, N 5.78 %, FA 0.7 %, HA 56.7 %, humin 24.1 %	0.5 kg m^{-2}	GC and GH	HS increased bell pepper tolerance to water stress conditions due to the reduction of leaf moisture loss and stimulation of root development	Water stress conditions	Qin et al., 2018
Sudan grass (*Sorghum vulgare* var. sudanen)	Humic substances 1: adding HA as K humate, 2: adding FA as K-fulvate, and 3: adding HA and FA.	Not specified	HA as K humate and FA as K fulvate were thoroughly mixed with the soil at the rate of 0.002 g kg^{-1} (equivalent to approximately 5 kg ha^{-1}) mixed with fine sand (3 g pot^{-1})	GH	The treatment of HA and FA gave the highest values of available NPK under the salinity levels	Under saline conditions and with/without foliar spray with Moringa leaf extract	Merwad, 2017
Onion (*Allium cepa* L.) cultivar Parachinar	Not specified	Not specified	0.5, 1, 1.5 mg L^{-1} (1, 2 and 3 kg ha^{-1})	FD	Application of humic at 2 kg ha^{-1} resulted in higher growth and yield for onion	---	Sajid et al., 2012
Cucumber (*Cucumis sativus* L.)	Humic acid, (Polymeric polyhydroxy acid % 85.5) produced by American Colloid Company under the trade name of Agro-Lig)	Not specified	1000 and 2000 mg L^{-1} (1.0 and 2.0 g kg^{-1} soil)	GH	Iron and Zn content of the leaf and stem tissues were increased under saline condition and humic acid treatments	Plants grown under saline conditions	Demir et al., 1999

Application Mode: Foliar application							
**Crop**	**Type of humic substances**	**Specification**	**Dosage**	**Condition**	**Effects**	**Observation**	**Reference**
Strawberry (Fragaria × ananassa)	Commercial products of humic acid	Density (1.1 kg dm^{-3}); pH 9.2; TOC (7.5 % w/w^{-1}); TON (0.1 % w w^{-1})	1000 mg L^{-1} (1.0 g L^{-1})	GH	Increased Si content in roots and increased Chroma values in fruits	---	Soppelsa et al., 2019
Dry bean (*Phaseolus vulgaris* L.)	Soluble humic acid as potassium-humate	Humic acid 80%, K_2O 11-13%	humic acid (500 ppm)	FD	The benefits of zinc foliar spray combined with humic acid and chitosan are inconsistent over both sowing dates, but may be more efficient in late-sown crops where heat stress coincided with the reproductive phase	---	Ibrahim and Ramadan, 2015
Faba bean (*Vicia faba* L. cultivar Sakha 4)	foliar application of nicotinamide and humic acid	Not specified	Nicotinamide at 5, 10, and 20 mg $L^{-1}$and/or humic acid at 5%	FD	Nicotinamide and/or humic acid had a positive effect on growth parameters, photosynthetic pigments, seed yield, and yield components as well as some biochemical constituents of the yielded faba bean seeds	---	Dawood et al., 2019
Coffe *(Coffea arabica* L. var. Catuaí vermelho I-144)	HA and FA commercial products extracted from leonardite.	Electrical conductivity (EC): 37.7 dS m^{-1}, pH: 9.7, E4/E6 ratio: 4.84, C: 38%, N: 0.9% for the HA sample. EC: 19.1 dS m^{-1}, pH: 5.6, E4/E6 ratio: 7.35, C: 39%, N: 0.35% for the FA sample	0, 10, 25, 50, 75 and 100 mg kg^{-1} (C-HA). 0, 0.2, 0.5 and 1g L^{-1} (C-FA)	GH	Foliar application of C-FA, instead soil application of C-HA, is a suitable practice to improve coffee seedlings growth and nutrition on Oxisol	---	Justi et al., 2019
Maize (*Zea mays* L. 'Qiangsheng No. 31')	Fulvic acid (FA)	The FA used in this study has properties similar to those of antitranspirants and auxin	2 g L^{-1}	FD	Soil superabsorbent polymer and foliar FA use under low rainfall conditions had little influence on crop water consumption but improved plant water-use-efficiency by enhancing photosynthesis and increasing kernel number	Deficit irrigation conditions	Yang et al., 2019
Chili pepper (*Capsicum annuum* var annuum)	Biomin (amino acid-based fertilize, Arbico-Co, Oro Valley,	Biomin a liquid, light brown material containing N 2%, Zn 2.5%, Mn 1.5%, Fe 1%,	Foliar spray in a concentration of 0.2%	GH	Higher values for leaf area, leaf number, chlorophyll index, root and shoot biomass, and leaf concentration of soluble sugars,	---	Souri and Sooraki, 2019

	AZ, USA). Humifolin (humic acid-based fertilizer; Tradecrop Co., Spain)	Mg 0.4%, Cu 0.4%.<br><br>Humifolin containing 42% organic compounds including 37% fulvic and humic acids, 7% vitamins, P 0.5%, Fe 0.28%, Zn 0.041% , Mn 0.0035%, Cu 0.0023%, Mg 0.0012%, B 0.0012%			N, K, Ca, and Zn, were attributed to foliar application of Biomin and Humifolin.		
Fenugreek (*Trigonella*)	humic acid in micronutrients solution	Sulfate form micronutrients solution was used with concentration: Fe 300 mg/l, Mn 100 mg/l, Zn 50 mg/l, and Cu 5 mg/l, added to EDTA and humic acid as a chelating agent.	All treatments received 20 m^3/fed compost, 100 kg/fed calcium superphosphate during soil preparation, 75 kg K_2O/fed as potassium sulfate and 200 kg ammonium sulfate which were added in two equal doses after 1 month from sowing and after 1 month from the first application	FD	The plant height increments were by 48 and 50% for sulfate form with compost tea, by 67 and 69% for humic acid form with compost tea and by 66% for EDTA form with compost tea for the first and second season, respectively	---	Ibrahim, 2019
Soybean [*Glycine max* (L.) Merr.]	Formulated humic product, Igniter (Innovative Crop Solutions, Radcliffe, IA), derived as a liquid extract from leonardite	Urea ammonium nitrate 9.0 %; P_20_5 9.0 %. K_20 3.0 %; Humic acid 1.21 %; Fulvic acid 0.78 %	Applied at 3.0-pint acre^{-1}	FD	Increased seed yield and income	---	Lenssen et al., 2019
Sage plant *(Salvia splendens)*	Not specified	Not specified	100, 500 and 1000 mg L^{-1} C_{HA}	GH	Humic acid resembles salinity effect. Suggest that sage is an ornamental plant sensitive to salinity and humic acid	Plants grown under saline conditions	Karimia et al., 2019
Grape (*Vitis vinifera* L.)	Commercial humic acids (BioHumusSol Company Ltd., Romania)	HA liquid form from vermicomposts 14.5 g humic substances L^{-1}: N-NO_3^- 19 ppm, N-NH_4^+ 104 ppm), P 22.5 ppm, K^+ 132 ppm, Ca^{2+} 39 ppm, Mg^{2+} 75 ppm, Na^+ 75 ppm	30000, 40000 and 50000 mg L^{-1} C_{HA} (30, 40 and 50 ml·L^{-1} HA)	FD	Humic acid may improve growth, yield, and berry quality attributes of grapevine.\	---	Popescu, 2018

Orange rubis®apricot (*Prunus armeniaca* L.)	Three commercial biostimulant products (Hendophyt®, Ergostim®, and Radicon®)	Hendophyt®: containing carbon 35%, organic nitrogen 4%, boron 0.25% Radicon®: humified organic substance 90% of total organic matter, C/N ratio 4; Ergostim®: N-acetiltiazolidin-4-carboxylic acid (AATC) 2.5%, and of triazolidinecarboxylic acid (ATC) 2%	Hendophyt®: 150 g 100 L^{-1} of water or 1500 mg L^{-1}; Radicon®: 500 g 100 L^{-1} of water or 5000 mg L^{-1}. Ergostim®: 200 ml 100 L^{-1} of water or 2000 mg L^{-1}.	FD	All biostimulant treatments gave higher values of antioxidant activity respect to the control	---	Tarantino et al., 2018
Brachiaria grass *(Brachiaria decumbens)*	The vermicompost used to obtain HAs was produced with a mixture of filter cake from sugarcane and cattle manure 5:1 (v/v)	Elemental composition analysis of HA, the values for total carbon, oxygen, nitrogen, hydrogen, and ash were 48.5%, 42.2%, 3.2%, 5.6%, and 0.51% (on a dry weight basis), respectively.	7.5, 15, 30 and 60 mg L^{-1} C_{HA}	GH	HAs used at 60 mg L^{-1} C applied at 15 DAE promoted increases of 44% in plant height and 196% in forage mass. After the cut, the optimum concentration was approximately 40 mg L^{-1} C	---	Pinheiro et al., 2018
Snap bean cv. Paulista	Humin Tech Company, Dosseldorf – Germany	Humic acid and K 85%, 12%; Fe 1%; pH 9-10.5. Density 0.55 kg. L^{-1}.	0.4 and 8 g L^{-1}	GH	Application of humic acid increased vegetative characters, pod yield and its characters, phosphorus, calcium, potassium, total protein percentage and total soluble phenols in green pods	---	Hanafy Ahmed et al., 2018
Wheat (*Triticum aestivum* L.)	The fulvic acid and humic acids were obtained by alkaline extraction of lignite coal	HA the coal was finely ground and 5% potassium hydroxide (KOH) solution was mixed with ground coal for 12 hr. FA the ground and powdered coal was treated with 10% nitric acid (HNO_3)	This plot received 12 liters of liquid fertilizer	FD	The liquid foliar fertilizer containing humic acid, NPK, zinc, sulfur and manganese and the foliar fertilizer has a significant effect on the biological yield of wheat. It was found the combination of these two fertilizers with urea greatly enhanced the yield of the wheat crop	---	Ahmad, Khan and Khattak, 2018
Spinach *(Spinacia oleracea* L. *varieties* ‘‘Balady and Firefly’’*)*	Humax fertilizer	Extract potassium humate (250 g L^{-1}), organic matter (200–250 g L^{-1}), fulvic acids (<20 g L^{-1}), amino acids (<20 g L^{-1}), nitrogen (< 50 g L^{-1}), phosphorus (P_2O_5,	2 - 4 g L^{-1} of Humax	FD	This treatment improved the quality of plant by increasing vitamin C content and reducing nitrite and oxalates contents	---	Alessa, et al., 2017

		< 50 g L^{-1}), potassium (K_2O, < 50 g L^{-1}) and trace elements (Mg, Fe, Zn, B, 10–15 g L^{-1})					
Safflower (*Carthamus tinctorius*)	Fulvic acid was prepared by Fanavari Sabz Shargh Co, Tehran, Iran.	Not specified	0.25 and 0.5 mg L^{-1} (0.5 and 1 kg ha^{-1})	FD	Higher seed yield and oil percentage	---	Moradi et al., 2017
Sugarcane (*Saccharum* spp.)	Humic acids extracted from vermicompost	The application of the biostimulant at a rate of 400 L ha^{-1}	20 mg L^{-1} C_{HA} of K^+ humate	FD	Foliar spray performed better than furrow application. Best result when applied at 60 days after emergence (37% of the stem yield). First and second ratoons, the productivity increases 5 and 24%	Combined use of humic acids and endophytic diazotrophic bacteria	Da Silva et al., 2017
Onion (*Allium cepa* L.)	The original commercial solution originating from leonardite (Nutriplant®, São Paulo, Brazil)	FA 10%, HS 90% and pH 4.0, originating from leonardite with C 34.4%, H 3.8% and N 2.3%.	Foliar pulverization: 1000 and 2000 mg L^{-1} Immersion method were: 10000 and 20000 mg L^{-1}	FD	Increase on onion yield, productivity, carbohydrates and proteins levels in bulbs, mineral nutrient accumulation resulted especially when highest doses of HS were added.	---	Bettoni et al., 2016a
Maize (*Zea mays* L.).	Residual solid remaining after the obtaining liquid humus (Liplant®) from cattle manure	M.O. 28.32 g.kg^{-1}, S.H. 19.04 g.kg^{-1}, H.A 8.94. g.kg^{-1}, A.F. 10.10 g.kg^{-1}, AH/AF 0.88 mS.cm^{-1}, C.E. 3.48 mS.cm^{-1}, pH (H_2O) 7.2	1:10, 1:20 and 1:30 (v:v)	FD	Positive effects on length and stem diameter, length of cobs without leaves, cobs fresh weight with and without leaves and on the yield of the number of cobs	---	García et al., 2016b
Winter wheat (*Triticum aestivum* L.)	Fulvic acid (FA) (Huitong Corp., Xinjiang, China)	Not specified	2 g L^{-1} (Total amount of spray 100 L ha^{-1})	FD	Spray of FA and abscisic acid both improved crop yield performance as well as water use efficiency	Exogenous abscisic acid (ABA) and fulvic acid (FA) application; and Water Deficit Conditions	Zhang et al., 2016
Maize (*Zea mays* L.)	Humic substances isolated from vermicompost	The inoculant was prepared by diluting 200 mL of bacterial in 800 mL of humic substances (pH 7.0). Final concentration of 50 mg. L^{-1} and final bacteria concentration of 2×10^8 cells. mL^{-1}	50 mg L^{-1}	FD	Combination of bacterium and humic acids significantly increased maize grain production at all urea-N rates (dry season) and less than 75 kg N-urea ha^{-1} (rain season)	Combined use with *H. seropedicae*	Canellas et al., 2015

Ryegrass (*Lolium Perenne* L.)	Humic acid from leonardite. Purchased from a Chinese company (Dalian Yano Agriculture Co., Dalian, China)	Containing: carbon (C), 61.2%; N, 3.13 g kg^{-1} dry matter; and P, 2.89 g kg^{-1} dry matter	100, 400, and 1000 mg L^{-1} C_{HA})	FD	Lower concentration of HA effective on N content increase and improving Lolium prenne "Speedygreen" visual quality.	---	Maibodi et al., 2015
Tomato (*Solanum lycopersicum* L.)	Extracted from vermicomposted cattle manure	Organic matter (dry weight basis) 36.2 g kg^{-1}, total humic substances 25.8 g kg^{-1}, humic acids 12.8 g kg^{-1}, fulvic acids 13.0 g kg^{-1}, pH 8.67, electrical conductivity 11.7 mS cm^{-1}, total nitrogen content 1.4 g kg^{-1}, total P content (as P_2O_5) 12.6 g kg^{-1}, and ash content 3%	Humates were diluted 20-fold in distilled water before application at 20 mg L^{-1} C_{HA}	GH and FD	Fruit yields increased (approximately 70%) significantly relative to control plants at the field by foliar spraying of PGPB and humates. Foliar spray (two application at field, was combined with substrate application at nursery	Combined use with *Herbaspirillum seropedicae*	Olivares et al., 2015
Ryegrass *(Lolium perenne* L.)	Humic acid was prepared from leonardite and purchased from a Chinese company (Dalian Yano Agriculture Co., Liaoning, China)	C 61.2%; N, 3.13 g kg^{-1} dry matter; and P, 2.89 g kg^{-1} dry matter	0, 100, 400, and 1000 mg L^{-1}	FD	Neither HA treatments nor mycorrhizal inoculation aff ected N and Fe contents of the leaves; P, K, and Zn concentrations improved by AM inoculation	Combined use with Arbuscular mycorrhizal (AM)	Nikbakht et al., 2014
Egyptian cotton (*Gossypium barbadense* L. cv. Giza 90)	Humic acid as a liquid was obtained from Seed Outlet in Agricultural Research Center, Giza, Egypt, "Super Canada"	Humic acid active 8%-folic acid active 1%, other organic materials 72.3% and neutral pH	0, 1 and 2%	GH	HA increased chemical constitutes related to salt tolerance either inorganic, N, P and K, while Na, Cl, Ca and Mg were decreased, or organic constitutes e.g. proline, total free amino acids, total sugars, total soluble phenols, chlorophyll a, b, total chlorophyll and total carotenoids	---	Ahmed, et al., 2013
Lettuce (*Lactuca sativa* var - *Black Seed Simpson*)	The isolated humates (alkaline extracts of vermicompost from cow manure)	The carbon content: 82.5 mmol C. The electrical conductivity was 5.81 mS·cm^{-1} and pH 8.73	150 mg L^{-1} C_{HA} (300 L·ha^{-1})	FD	21-days reduction in the production cycle (1.3 mmol C / L), while at 1.6 and 1.2 mmol C / L the reduction in the cycle was 15-days	---	Hernandez et al., 2013

Lilium (*Lilium* sp.)	Humic and fulvic acids	Not specified	Applications in bulb 0.5 and 1.0 % and for foliar applications were 0.050 and 0.1 %	GH	Increasing on anthocyanin content in lilium flowers. Soaking bulb method increased Zn, soluble sugar and µ-Amylase Enzyme concentration. Spraying method was more effective on anthocyanins contents increase.	---	Parandian; samavat, 2012
Pepper (*Capsicum annum* L.) cv. Demre sivrisi	HA was obtained from Cukurova Tarim Lombrico, Adana, Turkey	Solution contained 22% HA	10, 20, 30, and 40 ml L^{-1} providing 300, 600, 900, and 1200 mL/ha Humic acids (0.15, 0.3, 0.45 and 0.6 mg L^{-1} C_{HA})	GH	Soil and foliar HA treatment used to obtain higher fruit yield and fruit quality in organically grown pepper	---	Karakurt et al., 2009
Common Bean (*Phaseolus vulgaris* L.)	Acid substances HA (PHOSYN, Pocklington, U.K)	Not specified	2000 ml ha^{-1}	FD	Seed pretreatment with Zn or pretreatment of seeds + foliar spray of humic acid substances at three to six leaf stage significantly increased yield and yield components in common bean	---	Kaya et al., 2005
**Application Mode: Fertigation**							
**Crop**	**Type of humic substances**	**Specification**	**Dosage**	**Cond ition**	**Effects**	**Observation**	**Reference**
Citrus (*Citrus reticulata* cv. kinnow mandarin)	HA in liquid form (Lyallpur Chemicals & Fertilizers Ltd., Lahore).	Not specified	10% (80 mL)	FD	Plant growth and increased fruit yield. Also, physicochemical traits (ascorbic acid, total sugars)	Combined use with and without multi-nutrient foliar fertilizer “Micro Power”	Hameed et al., 2018
Melon (*Cucumis melo* ‘Galia’)	Potassium humate compound 83 % obtained from Union company for agricultural development.	Potassium humate compound 83 %	Humic acid (0, 7, 14, 21 L ha^{-1})	FD	The deleterious effect of salt stress on melon plant has been alleviated by the treatments with humic acid and boron.	Combined use with Boron and plant under saline conditions	Shalaby & El-Messairy, 2018
Roselle (*Hibiscus sabdariffa* L.)	Humic acid	Total humic extract 70%; humic acids 38%; polycarboxillic acid 32%; total organic	0 and 4kg ha^{-1}	FD	Humic acid application and mycorrhizal inoculation, especially using *G. intraradices*, showed the highest values of	Mycorrhizal inoculation (*Glomus versiforme*)	Fallahi, et al., 2017

		matter 70%; calcium oxide 1%; potassium oxide 10%			anthocyanins (56.9 mg.l^{-1})and vitamin C (2309 mg.100 g^{-1}) content.		
Tomato (*Solanum lycopersicum* L.)	humic acid, humicplus	Humic acid 12%	0.5 L per hectare or 0.25 mg L^{-1}	FD	Fertigation along with humic acid resulted in 20 percent fertilizer savings over fertigation alone	---	Suman et al., 2017
Spinach (*Spinacia oleraceae* L.),cv. 'Olbrzym zimowy'	Humic and fulvic acids (used in the form of the commercial concentrate Humistar, HU), manufactured by TRADECORP.	It is an extract obtained from leonardite, a natural form of humates, and contains 12% w/w of humic acids and 3% w/w of fulvic acids.	Pre-sowing HU application at doses of 0.0125, 0.05 and 0.2 cm^3 HU dm^{-3} of soil, fertigation at a dose of 0.0004% KIO_3	FD	The use of HU and fertigation with KIO_3 caused an increase of iodine content and iodine transfer factor (TF) from the soil to the plants		Smoleńet al., 2017
Banana (*Musa* sp.)	Humic substances extracted from a mineraloid rich in organic carbon known as leonardite	Humic acids 100 g kg^{-1}, fulvic acids 102g kg^{-1}, K 26.6 g kg^{-1}, and density 1.14 kg dm^{-3}	Reference dose (12.09 kg ha^{-1} cycle^{-1} or 6.045 mg L^{-1}) and 100%, 150%, 200%, 250% and 300%	FD	There was no effect of the humic substances doses evaluated	---	De Melo et al., 2016
Capsicum cv. 'California Wonder'	Humic acid, super potassium f-humate	potassium humate 75% and fulvic acid 15%	0.5 mg L^{-1} (1 kg ha^{-1})	FD	Fruit yield was 17–27 percent higher under combined fertigation over sole fertigation. Fertilizer use efficiency was higher in combined fertigation	With three levels of fertigation (100, 80 and 60% of recommended NPK)	Suman et al., 2016)
Potato (*Solanum tuberosum* L.)	Humic acid multinutrient fertilizer Grow Flow 45H (GF 45H) humic acid Supa Humus 26 (SH 26)	(GF 45H) N 14%, P 15%, K 11% and humus 1.2% (w/ v) and (SH 26) K 7% and humus 18% (w/v)	1.5 - 2.5 mg L^{-1} (3 - 5 L ha^{-1})	FD	Grow Flow 45H increased the N, P and K use efficiencies by 16.4%, 9.3% and 18.3% respectively over chemical fertilizer	100, 75, 50% of recommended dose of fertilizers (180 kg N, 90 kg (P_2O_5) and 100 kg (K_2O) per hectare	Selladurai and Purakayast h, 2016
Potato (*Solanum tuberosum* L.)	Humic Acid 86+6 % in black granule form (Ferzan Liquid Fertilizers, Theriad Energy Company, USA)	Huma K, Humic Acid 56 %, Fulvic Acid 30 %, Potassium 6 %	1500 mg L^{-1} (1.5 g L^{-1})	FD	HA application increased vegetative growth, tuber weight, yield, WUE, and tuber quality. Applying 1.5 g L−1 HA during vegetative growth and a 75 % ET water regime at S3 can increase potato production and tuber quality while reducing water use	The five irrigation treatments, based on crop evapotranspiration-ration (ETc)	Alenazi et al., 2016

Melon (*Cucumis melo* L. var. inodorus)	Vermicompost (2 500 kg ha^{-1}, 107 kg C ha^{-1}) was obtained from the stabilization of biological sewage sludge through the action of *Eisenia foetida* worms according	EC 1.8 µScm^{-1}, TOC 50.4 g kg^{-1} , C/N 25.0, pH 7.2	50 mg L^{-1} of humic carbon (90kg C ha^{-1})	FD	Improvement in soil fertility, suggested by the increase in soil organic carbon and nutrient (nitrate and macro- and microelements) contents and in soil microbial activities (hydrolytic and oxidative enzymes)	---	Masciandaro et al., 2014
Broccoli (*Broccoli oleracea* L. var. italica)	Humic substances were extracted from composted crop residues (rice straw, cotton stalks, and maize stalks)	Composition of Humic acids 14.8%, Fulvic acid 3.5 %, OM 71%, N 3.9%, P 0.13 %, K 3.22 %	60 mg L^{-1} (120 L ha^{-1})	FD	Application of HS increased total marketable yield and head diameter of broccoli as well as quality parameters. Higher nutrient concentrations were found in the broccoli heads and concentrations of plant-available nutrients in soil after harvesting were also higher.	Three rates of mineral fertilization (50%, 75%, and 100% of the recommended dose of NPK)	Selim and Mosa, 2012
Potato (*Solanum tuberosum* L.)	Combined fertilizers and humic substances were produced by Fertilizers Development Center, El-Delta Fertilizers Plant, Egypt	Composition of OM 68%, N 2.09%, P 0.15%, K 3.42%	30, 60 mg L^{-1} (60, 120 kg ha^{-1})	FD	Increasing humic substances application rates up to 120 kg ha^{-1} enhanced tubers yield quantity, starch content and total soluble solids.	---	Selim et al., 2009
Melon (*Cucumis melo*)	The humic substance used, from commercial product, complying with the requirements for organic food production in the European Union	total humic extract 22.9%, humic acids11.3% and fulvic acids 11.6%	15, 30 and 50 L ha^{-1} (7.5, 15, 25 mg L^{-1})	FD	The conventional fertilizer combined with 30 e 50 L ha^{-1} of humic substance produced the highest commercial yield	---	Pinto et al., 2008
Lepidium sativum and *Zea mays* L. var. Botanis	A compost obtained from a three months aerobic stabilization of a mixture of animal slurry and the organic	organic carbon19.9%, total Kjeldhal Nitrogen 2.10%, pH 7.99	30 and 60 mg L^{-1}	GC	stimulation of soil metabolic potential occurred using HS at low concentration as HC.	Soluble humic substances and saline solutions	Masciandaro, et al., 2002

	fraction of municipal residues						
**Application Mode: Immersion**							
**Crop**	**Type of humic substances**	**Specification**	**Dosage**	**Condition**	**Effects**	**Observation**	**Reference**
Maize (*Zea mays* L.*)*	HS were extracted from an Andosol - Ecuador (HS^{And}), Histosol - Brazil (HS^{Hist}) and vermicompost (HS^{Vc})	HS^{And} (%): C 48.58, H 6.24, N 3.69, O 41.49. HS^{Hist}(%): C 57.50, H 6.31, N 3.69, O 32.50. HS^{Vc} (%): C 42.68, H 6.88, N 3.08, O 47.36.	0 (-HS; control), 10, 20, 40 and 80 mg C(HS) L^{-1}.	GC and GH	Plant protection against salt stress by HS depends on the structures composing the HS. Having more labile structures is related to plant growth, whereas having more aromatic structures affects pigment production	Conditions of saline stress in maize growth	Pinos et al., 2019
Onion (*Allium cepa* L.)	Mineral Leonardite (Powhumus® Humintech GmbH - Germany)	organic carbon obtained by extraction 33%, grinding, precipitation and filtration, presenting a concentration of 8% potassium oxide (K_2O).	300 - 600 mg L^{-1} C_{HA} (0,3 g.L^{-1} - 0,6 g. L^{-1} humic acid)	GH and FD.	Growth promotion effect on onion early stages, improved bulb caliber and yield of a hybrid cultivar of 34%, increasing sugars and proteins.	Combined use with microalgae *Scenedesmus subspicatus*	Gemin et al., 2019
Grapes (*Vitis vinifera* L.)	Fulvic acid	Not specified	500, 1000, 5000, 10000 and 20000 mg L^{-1} C_{HA} (0.5, 1, 5, 10 and 20 mg/ml)	GC	Fulvic acid induces resistance to *B. cinereal* through the activation of phenylpropanoid pathway and can be used as a new activator of plant defense responses to control gray mold	Biological Control	Xu et al., 2019
Maize (*Zea mays* var. Dekal 7518)	HA were extracted from earthworm compost	Humic-like substances were extracted as described else- where (Canellas et al. 2002)	12, 42, 143 and 500 mg L^{-1} C_{HA}	GC	The population of *H. seropedicae* strain RAM10 colonizing root caps and Border cells (BC) increased in response to exogenous humic acids	Combined use with *H. seropedicae* strain RAM10	Canellas et al., 2017
Common beans (*Phaseolus vulgaris)*	Humic acid-like substances isolated from vermicompost	Vermicompost prepared using sugarcane filter cake. C 46%, N 5.7% and O 45%.	1 mL per seed containing 10^8 cells mL^{-1} and HA-like substances at 135 mg L^{-1}	GH	The recuperation of common beans after water stress was improved by co-inoculation with rhizobia and *H. seropedicae* in the presence of humic acid-like substances	Combined use with mixed rhizobia and *H. seropedicae* inoculations	Melo et al., 2017

Watercress (*Lepidium sativum* L.) and Chicory (*Cichorium intybus* L.)	Humic-like lignin from giant reed (*Arundo donax* L., AD)	Mass fraction (%) of lignin separated by sulfuric acid (34.10 SA), alkaline-H_2O_2 (6.33) and Klason (28.72). Average percentage (%) of solubility in water at pH 7 (4 SA) (85 OX), and T1ρH-NMR values (ms) (13.3 SA) (9.8 OX), for lignins separated by sulfuric acid (SA) and alkaline oxidation (OX)	1, 10 and 100 ppm of lignin organic carbon	GC	The water-soluble lignins may either directly function as GA on plant and seed development or positively perturb GA-related hormonal balances, thereby influencing GA- mediated physiological mechanisms	---	Savy et al., 2017
Algae (*Chlorella vulgaris*.)	Soil	1) Virgin chernozem. 2) Arable gray soil. 3) fallow chernozem. 4) virgin soddy-podzolic soil. 5) virgin gray soil. 6) 15-year-old fallow soddy-podzolic soil	0,001–0,003% humic acid (HA)	GC	Optimum range of HA concentrations for the growth of *Chlorella vulgaris* was: 0.001–0.003%. And the highest positive effect on total photosynthesis increment was observed for HAs preparations from fallow soddy-podzolic and virgin gray soils	---	Toropkina et al., 2017
Onion (*Allium cepa* L.)	Original commercial solution. Originating from Leonardite (Nutriplant®)	Humic substances containing fulvic acid 10%, humic substances 90% and pH 4.0, with C 34.4%, H 3.8%, and N 2.3%	0.0025- 0.0125 mg L^{-1} (5 - 25 mL L^{-1})	GH	Growth promotion of onion seedlings occurred between the concentrations of 17- and 20-mL L^{-1} of humic substances in the immersion solution	---	Bettoni et al., 2016b
(Eucalyptus urograndis)	Humic substance, from the commercial product - Fertiactyl®	Humic acids were extracted from 10 L of vermicompost with 100 L of 0.1 mol L^{-1} KOH solution overnight.	2.5, 5.0, 7.5 and 10mL L^{-1} (0.00125, 0.0025, 0.00375 and 0.005 mg L^{-1})	FD	The immersion of the tubes in solution with humic substance promotes the increase in post-planting survival and development of *Eucalyptus urograndis*	---	Da Silva et al., 2016
Soybean *(Glycine max* L.*)*	HA (Humus®; Broadtech Chemical International Co. Ltd., Inner Mongolia, China)	Potassium humate soluble granule 85% WSG	2.5 and 5 g L^{-1}	GH and FD	Soybean seed soaking in BTH + HA produced the highest activities of the testes of oxidative enzymes followed by BTH in the four soybean cultivars.	---	Abdel-monaimet al., 2011
Sugarcane (*Saccharum* hyb.)	Humic acids isolated from vermicompost produced from	C 48.5 %, H 5.5 %, N 3.2 %. The ash content was 0.5 % ± 0.001 %	20 mg L^{-1} C_{HA}	GH	Root growth was improved by 60 to 118 % in length and from 33 % to 233 % in surface area on sugarcane plant treatments	Combined use with *Herbaspirillum seropedicae*	Marques Júnior et al., 2008

	farmyard manure and red Californian earthworms				compared to control, with more pronounced effect in plants under heat treatment.		

REFERENCE

Abdel-monaim, M. F., Ismail, M. E., Morsy, K. M. (2011). Induction of Systemic Resistance of Benzothiadiazole and Humic Acid in Soybean Plants Against Fusarium Wilt Disease. *Mycobiology*, *39*(4), 290–298. https://doi.org/http://dx.doi.org/10.5941/MYCO.2011.39.4.290

Aguiar, N. O., Medici, L. O., Olivares, F. L., Dobbss, L. B., Torres-Netto, A., Silva, S. F., Novotny, E. H., Canellas, L. P. (2016). Metabolic profile and antioxidant responses during drought stress recovery in sugarcane treated with humic acids and endophytic diazotrophic bacteria. *Annals of Applied Biology*, *168*(2), 203–213. https://doi.org/10.1111/aab.12256

Aguiar, N. O., Olivares, F. L., Novotny, E. H., & Canellas, L. P. (2018). Changes in metabolic profiling of sugarcane leaves induced by endophytic diazotrophic bacteria and humic acids. *PeerJ*, *2018*(9), 1–28. https://doi.org/10.7717/peerj.5445

Ahmad, T., Khan, R., & Khattak, T. N. (2018). Effect of humic acid and fulvic acid based liquid and foliar fertilizers on the yield of wheat crop. *Journal of Plant Nutrition*, *41*(19), 2438–2445. https://doi.org/10.1080/01904167.2018.1527932

Ahmed, A. H. H., Darwish, E., Hamoda, S. A. F., Alobaidy, M. G. (2013). Effect of Putrescine and Humic Acid on Growth , Yield and ChemicalComposition of Cotton Plants Grown under Saline Soil Conditions. *American-Eurasian J. Agric. & Environ. Sci.*, (January). https://doi.org/10.5829/idosi.aejaes.2013.13.04.1965

Ahmed, A. H. H., Mohamed, H. F. Y., Orabi, I. O. A., El-Hefny, A. M. (2018). Influence of gamma rays, humic acid and sodium nitroprusside on growth, chemical constituents and fruit quality of snap bean plants under different soil salinity levels. *Bioscience Research*.

Alenazi, M., Wahb-Allah, M. A., Abdel-Razzak, H. S., Ibrahim, A. A., & Alsadon, A. (2016). Water Regimes and Humic Acid Application Influences Potato Growth, Yield, Tuber Quality and Water Use Efficiency. *American Journal of Potato Research*, *93*(5), 463–473. https://doi.org/10.1007/s12230-016-9523-7

Alessa, O., Najla, S., & Murshed, R. (2017). Improvement of yield and quality of two *Spinacia oleracea* L. varieties by using different fertilizing approaches. *Physiology and Molecular Biology of Plants*, *23*(3), 693–702. https://doi.org/10.1007/s12298-017-0453-8

Ali, A. Y. A., Ibrahim, M. E. H., & Zhou, G., Nimir, N. E. A., Jiao, X., Zhu, G., Elsiddig, A. M. I., Zhi, W., Chen, X., Lu, H. (2019). Ameliorative Effects of Jasmonic Acid and Humic Acid on Antioxidant Enzymes and Salt Tolerance of Forage Sorghum under Salinity Conditions. *Agronomy Journal*, *3108*, 3099–3108. https://doi.org/10.2134/agronj2019.05.0347

Bettoni, M. M., Mógor, Á. F., Kogerastki, J. F., & Pauletti, V. (2016b). Onion (*Allium cepa* L.) seedling growth using humic substances. *Idesia (Arica)*, (ahead), 0–0. https://doi.org/10.4067/s0718-34292016005000008

Bettoni, M. M., Mogor, Á. F., Pauletti, V., Goicoechea, N., Aranjuelo, I., & Garmendia, I. (2016a). Nutritional quality and yield of onion as affected by different application methods and doses of humic substances. *Journal of Food Composition and Analysis*, *51*, 37–44. https://doi.org/10.1016/j.jfca.2016.06.008

Canellas, L. P., Canellas, N. O. A., Soares, T. S., & Olivares, F. L. (2018). Humic Acids Interfere with Nutrient Sensing in Plants Owing to the Differential Expression of TOR. *Journal of Plant Growth Regulation*, *38*(1), 216–224. https://doi.org/10.1007/s00344-018-9835-6

Canellas, L. P., da Silva, S. F., Olk, D. C., & Olivares, F. L. (2015). Foliar application of plant growth-promoting bacteria and humic acid increase

maize yields. *Journal of Food, Agriculture & Environment*, *13*(1), 131–138. Recuperado de https://www.researchgate.net/publication/292574470

Canellas, L. P., & Olivares, F. L. (2017). Production of border cells and colonization of maize root tips by *Herbaspirillum seropedicae* are modulated by humic acid. *Plant and Soil*, *417*(1–2), 403–413. https://doi.org/10.1007/s11104-017-3267-0

Canellas, L. P., Olivares, F. L., Canellas, N. O. A., Mazzei, P., & Piccolo, A. (2019a). Humic acids increase the maize seedlings exudation yield. *Chemical and Biological Technologies in Agriculture*, *6*(1), 1–14. https://doi.org/10.1186/s40538-018-0139-7

Canellas, L. P., Olivares, F. L., Okorokova-Façanha, A. L., & Façanha, A. R. (2002). Humic acids isolated from earthworm compost enhance root elongation, lateral root emergence, and plasma membrane H+-ATPase activity in maize roots. *Plant Physiology*, *130*(4), 1951–1957. https://doi.org/10.1104/pp.007088

Canellas, N. O. A., Olivares, F. L., & Canellas, L. P. (2019b). Metabolite fingerprints of maize and sugarcane seedlings: searching for markers after inoculation with plant growth-promoting bacteria in humic acids. *Chemical and Biological Technologies in Agriculture*, *6*(1), 1–10. https://doi.org/10.1186/s40538-019-0153-4

Cieschi, M. T., Polyakov, A. Y., Lebedev, V. A., Volkov, D. S., Pankratov, D. A., Veligzhanin, A. A., Perminova, I. V., Lucena, J. J. (2019). Eco-friendly iron-humic nanofertilizers synthesis for the prevention of iron chlorosis in soybean (*Glycine max*) grown in calcareous soil. *Frontiers in Plant Science*, *10*(April), 1–17. https://doi.org/10.3389/fpls.2019.00413

Da-Silva, R. J., Ferreira Junior, J. M., Silva, F. A., Dos Santos, A. C. M., Lima, S. de O., & da Silva, R. R. (2016). Humic substances, purified MAP and hydrogel in the development and survival of eucalyptus urograndis. *Revista Brasileira de Engenharia Agricola e Ambiental*, *20*(7), 625–629. https://doi.org/10.1590/1807-1929/agriambi.v20n7p625-629

Da-Silva, S. F., Olivares, F. L., & Canellas, L. P. (2017). The biostimulant manufactured using diazotrophic endophytic bacteria and humates is effective to increase sugarcane yield. *Chemical and Biological Technologies in Agriculture*, *4*(1), 24. https://doi.org/10.1186/s40538-017-0106-8

Dawood, M. G., Abdel-baky, Y. R., El-awadi, M. E., Bakhoum, G. S. (2019). Enhancement quality and quantity of faba bean plants grown under sandy soil conditions by nicotinamide and / or humic acid application. *Bulletin of the National Research Centre*, *0*. https://doi.org/https://doi.org/10.1186/s42269-019-0067-0

De Aquino, A. M., Canellas, L. P., da Silva, A. P. S., Canellas, N. O., Lima, L. S., Olivares, F. L., Piccolo, A., Spaccini, R. (2019). Evaluation of molecular properties of humic acids from vermicompost by 13 C-CPMAS-NMR spectroscopy and thermochemolysis–GC–MS. *Journal of Analytical and Applied Pyrolysis*, *141*(June), 104634. https://doi.org/10.1016/j.jaap.2019.104634

De Azevedo, I. G., Olivares, F. L., Ramos, A. C., Bertolazi, A. A., & Canellas, L. P. (2019). Humic acids and *Herbaspirillum seropedicae* change the extracellular H+ flux and gene expression in maize roots seedlings. *Chemical and Biological Technologies in Agriculture*, *6*(1), 1–10. https://doi.org/10.1186/s40538-019-0149-0

De Melo, D. M., Coelho, E. F., Borges, A. L., Pereira, B. L. da S., & Campos, M. S. (2016). Agronomic performance and soil chemical attributes in a banana tree orchard fertigated with humic substances. *Pesquisa Agropecuária Tropical*, *46*(4), 421–428. https://doi.org/10.1590/1983-40632016v4642222

Demir, K., Gunes, A., Inal, A., & Alpaslan, M. (1999). Effects of humic acids on the yield and mineral nutrition of cucumber (*Cucumis sativus* L.) grown with different salinity levels. *Acta Horticulturae*, *492*, 95–103. https://doi.org/10.17660/actahortic.1999.492.11

Dobbss, L. B., Medici, L. O., Peres, L. E. P., Pino-Nunes, L. E., Rumjanek, V. M., Façanha, A. R., & Canellas, L. P. (2007). Changes in root development of Arabidopsis promoted by organic matter from oxisols. *Annals of Applied Biology*, *151*(2), 199–211. https://doi.org/10.1111/j.1744-7348.2007.00166.x

Ekin, Z. (2019). Integrated use of humic acid and plant growth promoting rhizobacteria to ensure higher potato productivity in sustainable agriculture. *Sustainability (Switzerland)*, *11*(12). https://doi.org/10.3390/su10023417

Façanha, A. R., Façanha, A. L. O., Olivares, F. L., Guridi, F., & Santos, G. De A., Velloso, A. C. X., Rumjanek, V. M., Brasil, F., Schripsema, J., Braz-Filho, R., De Oliveira, M. A., Canellas, L. P. (2002). Bioatividade de ácidos húmicos: Efeitos sobre o desenvolvimento radicular e sobre a bomba de prótons da membrana plasmática. *Pesquisa Agropecuaria Brasileira*, *37*(9), 1301–1310. https://doi.org/10.1590/s0100-204x2002000900014

Fallahi, H. R., Ghorbany, M., Aghhavani-Shajari, M., Samadzadeh, A., & Asadian, A. H. (2017). Qualitative response of roselle to planting methods, humic acid application, mycorrhizal inoculation and irrigation management. *Journal of Crop Improvement*, *31*(2), 192–208. https://doi.org/10.1080/15427528.2016.1269378

García, A. C., Santos, L. A., de Souza, L. G. A., Tavares, O. C. H., Zonta, E., Gomes, E. T. M., García-Mina, J. M., Berbara, R. L. L. (2016a). Vermicompost humic acids modulate the accumulation and metabolism of ROS in rice plants. *Journal of Plant Physiology*, *192*, 56–63. https://doi.org/10.1016/j.jplph.2016.01.008

García, A. C., Santos, L. A., Izquierdo, F. G., Rumjanek, V. M., Castro, R. N., dos Santos, F. S., de Souza, L. G. A., Berbara, R. L. L. (2014). Potentialities of vermicompost humic acids to alleviate water stress in rice plants (*Oryza sativa* L.). *Journal of Geochemical Exploration*, *136*, 48–54. https://doi.org/10.1016/j.gexplo.2013.10.005

García, A. C., Quintero, J. J. P., Balmori, D. M., López, R. H., & Izquierdo, F. G. (2016b). Efeitos no cultivo do milho de um extrato líquido humificado residual , obtido a partir de vermicomposto. *Revista Ciencias Técnicas Agropecuarias*, *25*(1), 38–43.

Gemin, L. G., Mógor, Á. F., De Oliveira Amatussi, J., & Mógor, G. (2019). Microalgae associated to humic acid as a novel biostimulant improving onion growth and yield. *Scientia Horticulturae*, *256*(January), 108560. https://doi.org/10.1016/j.scienta.2019.108560

Giro, V. B., Jindo, K., Vittorazzi, C., De Oliveira, R. S. S., Conceição, G. P., Canellas, L. P., & Olivares, F. L. (2016). Rock phosphate combined with phosphatesolubilizing microorganisms and humic substance for reduction of plant phosphorus demands from single superphosphate. *Acta Horticulturae*, *1146*, 63–68. https://doi.org/10.17660/ActaHortic.2016.1146.8

Hameed, A., Fatma, S., Wattoo, J. I., Yaseen, M., & Ahmad, S. (2018). Accumulative effects of humic acid and multinutrient foliar fertilizers on the vegetative and reproductive attributes of citrus (*Citrus reticulata* cv. kinnow mandarin). *Journal of Plant Nutrition*, *41*(19), 2495–2506. https://doi.org/10.1080/01904167.2018.1510506

Hernandez, O. L., Huelva, R., Guridi, F., Olivares, F. L., & Canellas, L. P. (2013). Humatos isolados de vermicomposto como promotores de crescimento em cultivo orgânico de alface. *Revista Ciencias Técnicas Agropecuarias*, *22*(1), 70–75.

Ibrahim, E. A., Ramadan, W. A. (2019). Effect of zinc foliar spray alone and combined with humic acid or/and chitosan on growth, nutrient elements content and yield of dry bean (*Phaseolus vulgaris* L.) plants sown at different dates. *Scientia Horticulturae*, *184*(February), 101–105. https://doi.org/10.1016/j.scienta.2014.11.010

Ibrahim, H. A. K. (2019). Effect of foliar application of compost water extract, humic acid, EDTA and micronutrients on the growth of fenugreek plants under sandy soil condition. *International Journal of Environmental Science and Technology*, *16*(12), 7799–7804. https://doi.org/10.1007/s13762-019-02311-9

Jindo, K., Soares, T. S., Peres, L. E. P., Azevedo, I. G., Aguiar, N. O., Mazzei, P., Spaccini, R., Piccolo, A., Olivares, F. L., Canellas, L. P. (2016). Phosphorus speciation and high-affinity transporters are influenced by humic substances. *Journal of Plant Nutrition and Soil Science*, *179*(2), 206–214. https://doi.org/10.1002/jpln.201500228

Justi, M., Morais, E. G., & Silva, C. A. (2019). Fulvic acid in foliar spray is more effective than humic acid via soil in improving coffee seedlings growth. *Archives of Agronomy and Soil Science*, *65*(14), 1969–1983. https://doi.org/10.1080/03650340.2019.1584396

Karakurt, Y., Unlu, H., Unlu, H., & Padem, H. (2009). The influence of foliar and soil fertilization of humic acid on yield and quality of pepper. *Acta Agriculturae Scandinavica Section B: Soil and Plant Science*, *59*(3), 233–237. https://doi.org/10.1080/09064710802022952

Karimian, Z., Samiei, L., & Nabati, J. (2019). Alleviating the salt stress effects in Salvia splendens by humic acid application. *Acta Scientiarum*

Polonorum, Hortorum Cultus, *18*(5), 73–82. https://doi.org/10.24326/asphc.2019.5.7

Kaya, M., Atak, M., Khawar, K. M., Çiftçi, C. Y., Özcan, S. (2005). Effect of Pre-Sowing Seed Treatment with Zinc and Foliar Spray of Humic Acids on Yield of Common Bean (*Phaseolus vulgaris* L .). *International journal of agriculture & biology*, 875–878. https://doi.org/1560–8530/2005/07–6–875–878

Kesba, H. H., & El-Beltagi, H. S. (2012). Biochemical changes in grape rootstocks resulted from humic acid treatments in relation to nematode infection. *Asian Pacific Journal of Tropical Biomedicine*, *2*(4), 287–293. https://doi.org/10.1016/S2221-1691(12)60024-0

Khan, R. U., Khan, M. Z., Khan, A., Saba, S., Hussain, F., & Jan, I. U. (2018). Effect of humic acid on growth and crop nutrient status of wheat on two different soils. *Journal of Plant Nutrition*, *41*(4), 453–460. https://doi.org/10.1080/01904167.2017.1385807

Lenssen, A. W., Olk, D. C., & Dinnes, D. L. (2019). Application of a Formulated Humic Product Can Increase Soybean Yield. *Cftm*, *5*(1), 0. https://doi.org/10.2134/cftm2018.07.0053

Li, X., Li, X., Han, B., Zhao, Y., Li, T., Zhao, P., & Yu, X. (2019). Improvement in lipid production in Monoraphidium sp. QLY-1 by combining fulvic acid treatment and salinity stress. *Bioresource Technology*, *294*(August), 122179. https://doi.org/10.1016/j.biortech.2019.122179

Mahoney, K. J., McCreary, C., Depuydt, D., & Gillard, C. L. (2017). Fulvic and humic acid fertilizers are ineffective in dry bean. *Canadian Journal of Plant Science*, *97*(2), 202–205. https://doi.org/10.1139/cjps-2016-0143

Maibodi, N. D. H., Kafi, M., Nikbakht, A., & Rejali, F. (2015). Effect of Foliar Applications of Humic Acid on Growth, Visual Quality, Nutrients Content and Root Parameters of Perennial Ryegrass (Lolium Perenne L.). *Journal of Plant Nutrition*, *38*(2), 224–236. https://doi.org/10.1080/01904167.2014.939759

Marques Júnior, R. B., Canellas, L. P., Da Silva, L. G., & Olivares, F. L. (2008). Promoção de enraizamento de microtoletes de cana-de-açúcar pelo uso conjunto de substâncias húmicas e bactérias diazotróficas endofíticas. *Revista Brasileira de Ciencia do Solo*, *32*(3), 1121–1128. https://doi.org/10.1590/S0100-06832008000300020

Masciandaro, G.; Ceccanti, B.; Ronchi, V.; Benedicto, S.; Howard, L. (2002). Humic substances to reduce salt effect on plant germination and growth. *Communications in Soil Science and Plant Analysis*, *33*(3–4), 365–378. https://doi.org/10.1081/CSS-120002751

Masciandaro, G., Peruzzi, E., Doni, S., & Macci, C. (2014). Fertigation with Wastewater and Vermicompost: Soil Biochemical and Agronomic Implications. *Pedosphere*, *24*(5), 625–634. https://doi.org/10.1016/S1002-0160(14)60048-5

McLean, K. L., Hunt, J. S., Stewart, A., Wite, D., Porter, I. J., & Villalta, O. (2012). Compatibility of a Trichoderma atroviride biocontrol agent with management practices of Allium crops. *Crop Protection*, *33*, 94–100. https://doi.org/10.1016/j.cropro.2011.11.018

Melo, A. da P., Olivares, F. L., Médici, L. O., Torres-Neto, A., Dobbss, L. B., & Canellas, L. P. (2017). Mixed rhizobia and *Herbaspirillum seropedicae* inoculations with humic acid-like substances improve water-stress recovery in common beans. *Chemical and Biological Technologies in Agriculture*, *4*(1), 1–9. https://doi.org/10.1186/s40538-017-0090-z

Merwad, A. M. A. (2017). Effect of humic and fulvic substances and Moringa leaf extract on Sudan grass plants grown under saline conditions. *Canadian Journal of Soil Science*, *97*(4), 703–716. https://doi.org/10.1139/cjss-2017-0050

Monda, H., Cozzolino, V., Vinci, G., Drosos, M., Savy, D., & Piccolo, A. (2018). Molecular composition of the Humeome extracted from different green composts and their biostimulation on early growth of maize. *Plant and Soil*, *429*(1–2), 407–424. https://doi.org/10.1007/s11104-018-3642-5

Moradi, P., Pasari, B., & Fayyaz, F. (2017). The effects of fulvic acid application on seed and oil yield of safflower cultivars. *Journal of Central European Agriculture*, *18*(3), 584–597. https://doi.org/10.5513/JCEA01/18.3.1933

Nardi, S., Muscolo, A., Vaccaro, S., Baiano, S., Spaccini, R., & Piccolo, A. (2007). Relationship between molecular characteristics of soil humic fractions and glycolytic pathway and krebs cycle in maize seedlings. *Soil Biology and Biochemistry*, *39*(12), 3138–3146. https://doi.org/10.1016/j.soilbio.2007.07.006

Nikbakht, A., Pessarakli, M., Daneshvar-Hakimi-Maibodi, N., Kafi, M. (2014). Perennial ryegrass growth responses to mycorrhizal infection and humic acid treatments. *Agronomy Journal*, *106*(2), 585–595. https://doi.org/10.2134/agronj2013.0275

Nikbakht, A., Kafi, M., Babalar, M., Xia, Y. P., Luo, A., & Etemadi, N. A. (2008). Effect of humic acid on plant growth, nutrient uptake, and postharvest life of gerbera. *Journal of Plant Nutrition*, *31*(12), 2155–2167. https://doi.org/10.1080/01904160802462819

Noroozisharaf, A., & Kaviani, M. (2018). Effect of soil application of humic acid on nutrients uptake, essential oil and chemical compositions of garden thyme (Thymus vulgaris L.) under greenhouse conditions. *Physiology and Molecular Biology of Plants*, *24*(3), 423–431. https://doi.org/10.1007/s12298-018-0510-y

Nunes, R. O., Domiciano, G. A., Alves, W. S., Melo, A. C. A., Nogueira, F. C. S., Canellas, L. P., Olivares, F. L., Zingali, R. B., Soares, M. R. (2019). Evaluation of the effects of humic acids on maize root architecture by label-free proteomics analysis. *Scientific Reports*, *9*(1), 1–11. https://doi.org/10.1038/s41598-019-48509-2

Olivares, F. L., Aguiar, N. O., Rosa, R. C. C., Canellas, L. P. (2015). Substrate biofortification in combination with foliar sprays of plant growth promoting bacteria and humic substances boosts production of organic tomatoes. *Scientia Horticulturae*, *183*(1), 100–108. https://doi.org/10.1016/j.scienta.2014.11.012

Ozfidan-Konakci, C., Yildiztugay, E., Bahtiyar, M., & Kucukoduk, M. (2018). The humic acid-induced changes in the water status, chlorophyll fluorescence and antioxidant defense systems of wheat leaves with cadmium stress. *Ecotoxicology and Environmental Safety*, *155*(February), 66–75. https://doi.org/10.1016/j.ecoenv.2018.02.071

Parandian F., & Samavat S. (2012). Effects of Fulvic and Humic acid on Anthocyanin, soluble Sugar, Amylase Enzyme and some micronurient elements in Lilium. *International Research Journal of Applied and Basic Sciences*, *3*(5), 924–929. Recuperado de http://www.irjabs.com

Pinheiro, P. L., Passos, R. R., Peçanha, A. L., Canellas, L. P., Olivares, F. L., & de Sá Mendonça, E. S. (2018). Promoting the growth of Brachiaria decumbens by humic acids (HAs). *Australian Journal of Crop Science*, *12*(7), 1114–1121. https://doi.org/10.21475/ajcs.18.12.07.PNE1038

Pinos, N. Q., Berbara, R. L. L., Tavares, O. C. H., & García, A. C. (2019). Different Structures in Humic Substances Lead to Impaired Germination but Increased Protection against Saline Stress in Corn. *Communications in Soil Science and Plant Analysis*, *50*(17), 2209–2225. https://doi.org/10.1080/00103624.2019.1659294

Pinto, J. M., Gava, C. A. T., Lima, M. A. C., Silva, A. F., & Resende, G. M. de. (2008). Cultivo orgânico de meloeiro com aplicação de biofertilizantes e doses de substância húmica via fertirrigação. *Revista Ceres*, *55*(4), 280–286.

Popescu, G. C., & Popescu, M. (2018). Yield, berry quality and physiological response of grapevine to foliar humic acid application. *Bragantia*, *77*(2), 273–282. https://doi.org/10.1590/1678-4499.2017030

Prado, M. R. V., Weber, O. L. dos S., Moraes, M. F. de, Santos, C. L. R. dos, Tunes, M. S., & Ramos, F. T. (2016). Humic Substances on Soybeans Grown Under Water Stress. *Communications in Soil Science and Plant Analysis*, *47*(21), 2405–2413. https://doi.org/10.1080/00103624.2016.1243715

Qin, K., & Leskovar, D. I. (2018). Lignite-derived humic substances modulate pepper and soil-biota growth under water deficit stress. *Journal of Plant Nutrition and Soil Science*, *181*(5), 655–663. https://doi.org/10.1002/jpln.201800078

Qin, K., & Leskovar, D. I. (2019). Deficit irrigation and humic substances residuals affected watermelon yield and soil properties. *Acta Horticulturae*, *1253*, 381–388. https://doi.org/10.17660/ActaHortic.2019.1253.50

Qin, Kuan, Dong, X., Jifon, J., & Leskovar, D. I. (2019). Rhizosphere microbial biomass is affected by soil type, organic and water inputs in a bell pepper system. *Applied Soil Ecology*, *138*(November 2018), 80–87. https://doi.org/10.1016/j.apsoil.2019.02.024

Rodda, M. R. C., Canellas, L. P., Façanha, A. R., Zandonadi, D. B., Guerra, J. G. M., Almeida, D. L., & Santos, G. A. (2006). Estímulo no crescimento e na hidrólise de ATP em raízes de alface tratadas com humatos de vermicomposto. I - Efeito da concentração. *Revista Brasileira de Ciência do Solo*. Recuperado de http://www.redalyc.org/articulo.oa?id=180214057005%0AComo

Rosa, S. D., Silva, C. A., & Maluf, H. J. G. M. (2018). Wheat nutrition and growth as affected by humic acid-phosphate interaction. *Journal of Plant Nutrition and Soil Science*, *181*(6), 870–877. https://doi.org/10.1002/jpln.201700532

Rosa, S. D., Silva, C. A., & Maluf, H. J. G. M. (2019). Phosphorus availability and soybean growth in contrasting Oxisols in response to humic acid concentrations combined with phosphate sources. *Archives of Agronomy and Soil Science*, *00*(00), 1–16. https://doi.org/10.1080/03650340.2019.1608527

Sajid, M., Rab, A., Shah, S. T., Jan, I., Haq, I., Haleema, B., Zamin, M., Alam, R., Zada, H. (2012). Humic acids affect the bulb production of onion cultivars. *African Journal of Microbiology Research*, *6*(28), 5769–5776. https://doi.org/10.5897/ajmr11.1643

Savy, D., Canellas, L., Vinci, G., Cozzolino, V., & Piccolo, A. (2017). Humic-Like Water-Soluble Lignins from Giant Reed (Arundo donax L.) Display Hormone-Like Activity on Plant Growth. *Journal of Plant Growth Regulation*, *36*(4), 995–1001. https://doi.org/10.1007/s00344-017-9696-4

Schoebitz, M., López, M. D., Serri, H., Aravena, V., Zagal, E., & Roldán, A. (2019). Characterization of Bioactive Compounds in Blueberry and Their Impact on Soil Properties in Response to Plant Biostimulants. *Communications in Soil Science and Plant Analysis*, *50*(19), 2482–2494. https://doi.org/10.1080/00103624.2019.1667374

Seenivasan, N., & Senthilnathan, S. (2018). Effect of humic acid on Meloidogyne incognita (Kofoid & White) Chitwood infecting banana (*Musa* spp.). *International Journal of Pest Management*, *64*(2), 110–118. https://doi.org/10.1080/09670874.2017.1344743

Selim, E. M., & Mosa, A. A. (2012). Fertigation of humic substances improves yield and quality of broccoli and nutrient retention in a sandy soil. *Journal of Plant Nutrition and Soil Science*, *175*(2), 273–281. https://doi.org/10.1002/jpln.201100062

Selim, E. M., Mosa, A. A., & El-Ghamry, A. M. (2009). Evaluation of humic substances fertigation through surface and subsurface drip irrigation systems on potato grown under Egyptian sandy soil conditions. *Agricultural Water Management*, *96*(8), 1218–1222. https://doi.org/10.1016/j.agwat.2009.03.018

Selladurai, R., & Purakayastha, T. J. (2016). Effect of humic acid multinutrient fertilizers on yield and nutrient use efficiency of potato. *Journal of Plant Nutrition*, *39*(7), 949–956. https://doi.org/10.1080/01904167.2015.1109106

Shalaby, O. A. E. ., & El-Messairy, M. M. (2018). Humic acid and boron treatment to mitigate salt stress on the melon plant. *Acta Agriculturae Slovenica*, *111*(2), 349–356. https://doi.org/10.14720/aas.2018.111.2.10

Smoleń, S., Ledwożyw-Smoleń, I., Sady, W. (2017). Iodine biofortification of spinach by soil fertigation with additional application of humic and fulvic acids. *New Zealand Journal of Crop and Horticultural Science*, *45*(4), 233–250. https://doi.org/10.1080/01140671.2017.1314307

Soppelsa, S., Kelderer, M., Casera, C., Bassi, M., Robatscher, P., Matteazzi, A., & Andreotti, C. (2019). Foliar applications of biostimulants promote growth, yield and fruit quality of strawberry plants grown under nutrient limitation. *Agronomy*, *9*(9), 1–22. https://doi.org/10.3390/agronomy9090483

Souri, M. K., & Sooraki, F. Y. (2019). Benefits of organic fertilizers spray on growth quality of chili pepper seedlings under cool temperature. *Journal of Plant Nutrition*, *42*(6), 650–656. https://doi.org/10.1080/01904167.2019.1568461

Suman, S., Spehia, R. S., & Sharma, V. (2016). Productivity of capsicum as influenced by fertigation with chemical fertilizers and humic acid. *Journal of Plant Nutrition*, *39*(3), 410–416. https://doi.org/10.1080/01904167.2015.1069338

Suman, S., Spehia, R. S., & Sharma, V. (2017). Humic acid improved efficiency of fertigation and productivity of tomato. *Journal of Plant Nutrition*, *40*(3), 439–446. https://doi.org/10.1080/01904167.2016.1245325

Tapia, Y., Casanova, M., Castillo, B., Acuña, E., Covarrubias, J., Antilén, M., & Masaguer, A. (2019). Availability of copper in mine tailings with humic substance addition and uptake by Atriplex halimus. *Environmental Monitoring and Assessment*, *191*(11). https://doi.org/10.1007/s10661-019-7832-2

Tarantino, A., Lops, F., Disciglio, G., & Lopriore, G. (2018). Effects of plant biostimulants on fruit set, growth, yield and fruit quality attributes of

‘Orange rubis®’ apricot (*Prunus armeniaca* L.) cultivar in two consecutive years. *Scientia Horticulturae*, *239*(February), 26–34. https://doi.org/10.1016/j.scienta.2018.04.055

Toropkina, M. A., Ryumin, A. G., Kechaikina, I. O., & Chukov, S. N. (2017). Effect of humic acids on the metabolism of *Chlorella vulgaris* in a model experiment. *Eurasian Soil Science*, *50*(11), 1294–1300. https://doi.org/10.1134/S1064229317110126

Xu, D., Deng, Y., Xi, P., Yu, G., Wang, Q., Zeng, Q., Jiang, Z., Gao, L. (2019). Fulvic acid-induced disease resistance to *Botrytis cinerea* in table grapes may be mediated by regulating phenylpropanoid metabolism. *Food Chemistry*, *286*(January), 226–233. https://doi.org/10.1016/j.foodchem.2019.02.015

Yang, W., Guo, S., Li, P., Song, R., & Yu, J. (2019). Foliar antitranspirant and soil superabsorbent hydrogel affect photosynthetic gas exchange and water use efficiency of maize grown under low rainfall conditions. *Journal of the Science of Food and Agriculture*, *99*(1), 350–359. https://doi.org/10.1002/jsfa.9195

Zandonadi, D. B., Matos, C. R. R., Castro, R. N., Spaccini, R., Olivares, F. L., & Canellas, L. P. (2019). Alkamides : a new class of plant growth regulators linked to humic acid bioactivity. *Chemical and Biological Technologies in Agriculture*, *4*, 1–12. https://doi.org/10.1186/s40538-019-0161-4

Zhang, H., Xie, S., Bao, Z., Tian, H., Carranza, E. J. M., Xiang, W., Yao, L., Zhang, H. (2019). Underlying dynamics and effects of humic acid on selenium and cadmium uptake in rice seedlings. *Journal of Soils and Sediments*. https://doi.org/10.1007/s11368-019-02413-4

Zhang, X., Zhang, X., Liu, X., Shao, L., Sun, H., Chen, S. (2016). Improving Winter Wheat Performance by Foliar Spray of ABA and FA Under Water Deficit Conditions. *Journal of Plant Growth Regulation*, *35*(1), 83–96. https://doi.org/10.1007/s00344-015-9509-6
